# Progress in the Preclinical Discovery and Clinical Development of Class I and Dual Class I/IV Phosphoinositide 3-Kinase (PI3K) Inhibitors 

**DOI:** 10.2174/092986711796011229

**Published:** 2011-06

**Authors:** S.J Shuttleworth, F.A Silva, A.R.L Cecil, C.D Tomassi, T.J Hill, F.I Raynaud, P.A Clarke, P Workman

**Affiliations:** 1 Karus Therapeutics Ltd., Southampton Science Park, 2 Venture Road, Southampton, SO16 7NP, UK; 2 Cancer Research UK Cancer Therapeutics Unit, Division of Cancer Therapeutics, The Institute of Cancer Research, Haddow Laboratories, 15 Cotswold Road, Sutton, Surrey SM2 5NG, UK

**Keywords:** PI3K, inhibitor, p110α, p110β, p110δ, p110γ, mTOR, cancer, inflammation, cardiovascular.

## Abstract

The phosphoinositide 3-kinases (PI3Ks) constitute an important family of lipid kinase enzymes that control a range of cellular processes through their regulation of a network of signal transduction pathways, and have emerged as important therapeutic targets in the context of cancer, inflammation and cardiovascular diseases. Since the mid-late 1990s, considerable progress has been made in the discovery and development of small molecule ATP-competitive PI3K inhibitors, a number of which have entered early phase human trials over recent years from which key clinical results are now being disclosed. This review summarizes progress made to date, primarily on the discovery and characterization of class I and dual class I/IV subtype inhibitors, together with advances that have been made in translational and clinical research, notably in cancer.

## 1 INTRODUCTION

The PI3K superfamily has, over the past 15 years, become one of the most extensively studied classes of therapeutic targets in small molecule drug discovery, particularly in oncology [1-4]. Four distinct PI3K subfamilies exist – commonly referred to as class I, II, III and IV – based upon their substrate specificities, primary structures, modes of regulation and domain content. Of these, it is the class I isoforms, p110α, p110β, p110δ and p110γ, together with the class IV PI3K-related kinase (PIKK), mTOR [5,6], which have been the most intensively examined targets in the small molecule therapeutic arena, and which form the principal subject of this present discussion.

## 2 CLASS I PI3K ISOFORMS AND mTOR IN CHRONIC DISEASE: POTENTIAL FOR SMALL MOLECULE INHIBITORS

The majority of small molecule discovery research in the PI3K field has, to date, centred on class I inhibitors for the treatment of cancer. The key mechanistic rationale for this stems from the class I PI3K/AKT pathway being dysregulated in a tissue-diverse range of tumours [1-4, 7,8]. The phosphatase PTEN, the negative regulator of PI3K, is one of the most commonly mutated proteins in human malignancy [9]. Furthermore, the gene encoding for the p110α subunit, *PIK3CA*, is amplified, overexpressed and frequently mutated in many cancers [10]; critically, these mutations have been shown to reduce cellular dependence on growth factors, to attenuate apoptosis, and to facilitate tumour invasiveness. Additionally, a greater understanding of the specific roles of the p110α and p110β isoforms in tumourigenesis has recently been established: it has been shown that p110α is critical for the growth of tumours driven by *PIK3CA* mutations as well as oncogenic receptor tyrosine kinases and RAS, whilst p110β is the principal isoform involved in mediating PTEN-deficient tumourigenesis [11-13]. In addition, p110δ has also emerged as a key therapeutic target for haematological malignancies [14,15], notably acute myeloid leukaemia (AML), and there is also some evidence that this isoform is upregulated in melanoma and breast cancer, and is overexpressed in neuroblastoma [4]. Furthermore, there is potential for all the class I PI3Ks to be activated in cancer cells through mutation of the p85 regulatory subunits. Consequently, inhibition of class IA PI3Ks – p110α, p110β and p110δ – represents an important strategy for the development of novel cancer therapeutics, and, moving forward, is anticipated to have a significant impact on the discovery and development of new personalized medicines in the oncology setting.

In addition to PTEN-null tumours, p110β has been pursued as a target for antithrombotic therapy [16], and there is also growing evidence that p110β inhibitors could have significant therapeutic potential in autoimmune diseases [17-19]. Furthermore, p110γ has been reported to play an important role in mast cell, eosinophil and neutrophil function [20]. Interestingly, the p110γ isoform was the first of the PI3K enzyme family for which a liganded crystal structure was resolved [21], and it has since been the subject of a number of small molecule R&D activities [4]. However, at the time of writing, there are no examples of p110γ-specific inhibitors to have entered clinical development. By contrast, the p110δ subtype, which has also been shown to play a central function in the recruitment and activation of a range of immune and inflammatory cells [22-24], has become a hotly pursued target in small molecule drug discovery circles. Co-crystal structures of this isoform were recently resolved [25], and there are several p110δ-targeted inhibitors that are currently in preclinical development – with two having now entered early phase clinical studies – for the treatment of haematological cancer and immune-inflammatory disorders [4,26].

Finally, there has been significant recent progress made in the discovery of new small molecules that target the PIKK sub-family member, mTOR. This protein was originally discovered in the 1990s, when the mechanism of action of rapamycin, a macrolide-based natural product with immunosuppressant activity, was elucidated [27]. Rapamycin and derivatives thereof bind with high afﬁnity to the immunophilin FK506-binding protein-12 (FKBP12), forming a complex that selectively inhibits mTORC1 downstream signalling to elements involved in growth control, and they have since been evaluated as agents for the treatment of solid tumours [4]. In addition, recent progress been made in targeting the ATP-binding site of mTOR with small molecule inhibitors that exhibit anti-tumour activity. Of particular significance to this present review, however, is the discovery and development of a number of small molecules that dually inhibit class I PI3Ks – particularly p110α – together with mTOR for the treatment of cancer, and these are discussed below. Inhibitors of class IV PI3Ks involved in DNA repair are also of interest in cancer, but will not be covered here

## 3 ISOFORM-SELECTIVE PI3K INHIBITORS: HISTORICAL LANDSCAPE AND RECENT PROGRESS

Since the discovery of LY294002 **1** [28] and the elucidation of the mechanism of action of the natural product Wortmannin **2 **[29], both of which display activity against the class I PI3K isoforms, considerable progress has been made in the development of a plethora of structurally-diverse inhibitors that possess distinct subtype selectivity profiles. The properties of a number of these compounds – including some that have advanced into clinical development – have been reviewed extensively elsewhere [4, 30-32]. Examples include the reversible, ATP-competitive p110α/pan-class I selective inhibitors GDC-0941, **3** [33,34], XL147, **4** [35], GSK1059615, **5** [36], and ZSTK474, **6** [37]; the irreversible p110α inhibitor PX-866, **7** [29]; the p110δ-selective inhibitor CAL-101, **8** [38]; the dual pan-class I/mTOR inhibitors SF1126, **9** [39], NVP-BEZ235, **10** [40], XL765, **11** [41] and GSK1059615, **12** [36]; the dual p110δ/γ inhibitor TG100115, **13** [29]; and the p110β-selective TGX-221, **14** [42]. Clinical data for several of these agents are summarized in Section 5. Additional small molecule PI3K inhibitors reported to be in pre-clinical discovery or development as of mid-2009 include compounds **14** through **36** (Table **1**), the biochemical, cellular and pharmacological properties of which have been chronicled previously [4], and which will not be discussed here. Rather, we illustrate the impact of structure-based drug design and focus on providing details of compounds disclosed in the primary and patent literature since that time. The chemical structures of these more recent compounds are listed in Table **2**.

### 3.1 Pan-Class I, Dual Pan-Class I/mTOR and Dual p110α/mTOR Inhibitors

#### 3.1.1 Impact of Co-Crystal Structure Elucidation on the Design of Novel Small Molecule PI3K Inhibitors

In considering the overall progress on the fascinating journey from early chemical tools to potent, selective and drug-like PI3K inhibitors that are now in the clinic, the exploitation of X-ray crystal structures of p110 catalytic domains to enable structure-based design has been especially valuable [3]. As an example, Fig. (**1**) shows the co-crystal structure of the clinical pan-class I PI3K drug GDC-0941 **3** bound to human p110γ. Overall there is a snug fit of the inhibitor in the ATP binding site and key features of the tridentate binding mode are: 1) the use of the morpholine oxygen to form a hydrogen bonding interaction with the amide of the hinge region Val 882 that is also bound by the adenine in ATP (left-hand side), representing an example of the privileged aryl morpholine structure that is seen commonly in many PI3K inhibitors; 2) the indazole moiety (right-hand side) which fits deep in the so-called affinity pocket and in which the two indazole nitrogen atoms form hydrogen bonds with the hydroxyl group of Tyr 867 and the carboxylate of Asp 841; and 3) the 4-methanesulfonyl–piperazin-1-ylmethyl group (projecting out of the plane, centre) that points to the solvent channel and has a solubilising function, but also makes additional binding interactions with the protein through the piperazine ring lying close to the side chain of Met 804 and the sulfonyl group forming hydrogen bonds with Ala 805 and Lys 802 at the mouth of the ATP pocket. Furthermore, the thienopyrimidine core is sandwiched between Met 953 and Ile 963 (forming the floor of the ATP pocket) and the side chains of Met 804, Trp 812 and Ile 831 (which form the ceiling of the ATP site) [3,33, 34]. 

Structural biology insights are now facilitating both the design of new PI3K inhibitors with distinct isoform selectivities, and the interpretation of the binding properties of existing small molecules inhibitors of the superfamily. As an example of the impact of crystal structures and the potential for structure-based design, most PI3K inhibitors bind to p110γ in a flat orientation, in the same plane as that adopted by the adenine of ATP; in contrast, an inducible and conformationally flexible specificity pocket is involved in the selectivity of compounds that act preferentially on p110δ, involving amino acid residues that are distal to the ATP binding site and that are more variable between p110 isoforms [106,107]. The p110δ isoform is more conformationally flexible than p110β and p110γ, and inhibitors preferring p110δ adopt a propeller shape which allows them to induce and access the specificity pocket. The specificity pocket appears to be more easily inducible and accessible in p110δ compared to p110γ and, moreover, it is predicted that p110α will be unable to undergo this conformational rearrangement.

#### 3.1.2 Recent Examples of New ATP-Competitive PI3K Inhibitors

As mentioned above, we have previously chronicled details of the *in vitro* and *in vivo* properties of a plethora of diverse small molecule PI3K inhibitors developed up to mid-2009 (Table **1**, compounds **14-36**) [4]. A number of those compounds are now in clinical development, and are discussed in further detail below. Over the past 18 months, the field has continued to develop at a substantial pace, with many examples of novel ATP-competitive inhibitors having been disclosed in the literature during that time. There has, in particular, been significant progress made in the development of pan-class I/mTOR dual inhibitors, and of class I inhibitors with distinct isoform selectivity profiles. The dominant therapeutic focus continues to be cancer, although inhibitors of other isoforms – particularly p110δ and p110γ – with utility in the treatment of immune-inflammatory diseases – have also been developed. One significant development in the PI3K arena is the emerging, compelling evidence that targeting of p110β with selective small molecule inhibitors may provide therapeutic benefit in the treatment of autoimmune diseases [19], as well as in the treatment of specific (PTEN-null) tumour types [12]. This is a relatively unexplored area, however, and p110β inhibitors are scarce. However, we expect the discovery and development of such inhibitors to be the subject of increasing focus over the coming years.

Table **2** lists the chemical structures of compounds **37-87**, which represent a new series of small molecule PI3K inhibitors that have been reported in the literature during the last 18 months. These are predominantly reversible, ATP-competitive inhibitors, and many feature the aryl-morpholine structural unit, an established hinge-targeted structural motif. It is clear in these examples, however, that subtle structural modifications can lead to quite dramatic changes to class I and IV subtype selectivity.

Yuan and co-workers have developed a series of potent demethoxyviridin derivatives which display significantly improved *in vivo* stability compared with demethoxyviridin. It was observed that esterification of the C1 position of demethoxyviridin led to an increase in serum half-life to two hours from 26 minutes; furthermore, conjugate addition with glycine furnished a derivative, **37**, whose half-life was 218 minutes, and which displayed an IC_50_ of 44nM [62].

Researchers at the University of Auckland have disclosed results from their virtual screening approaches to the discovery of new chemical entities targeting PI3K activity [63]. Following *in silico* screening of the ZINC compound database, which comprises 2.5 million compounds, a total of 89 new chemotypes were identified; biochemical screening subsequently led to the discovery of seven new compounds displaying activity between 1 and 100µM of which **38** was the most active, displaying pan-PI3K inhibition with an IC_50 _of 0.9µM, 3µM, 0.9µM and 4µM against p110α, β, δ and γ respectively.

Gilbert and colleagues at Wyeth have reported the discovery of two structurally-related series based on purine [3,4-*d*]pyrimidines and pyrazolo [3,4-*d*]pyrimidines, exemplified by **39** and **40 **respectively [64]. Interestingly, subtle structural changes were seen to lead to dramatic alterations in subtype selectivity: compound **39** had an IC_50 _of 58nM against p110α, and displayed good selectivity over p110γ and mTOR, whereas **40 **showed more dual p110α/mTOR activity (IC_50 _= 32nM and 83nM respectively), though with high selectivity over p110γ (IC_50 _= 2367nM).

Venkatesan and co-workers at Wyeth have outlined the development of imidazolopyrimidine derivatives exhibiting selective inhibition of both the class I isoforms and mTOR [65]. An example of such a compound is **41**, which had an IC_50_ of 16nM and 265nM against p110α and p110γ respectively, but was inactive against mTOR. In a separate report, the same team also described the design, synthesis, and characterization of the highly potent bismorpholino-1,3,5-triazine derivative **42 **(PKI-587), a potent dual class I PI3K/mTOR inhibitor [66]. This compound was seen to inhibit cell survival and proliferation, and to increase apoptosis *in vitro *and* in vivo*. PKI-587 also exerted potent anti-tumour efficacy in preclinical subcutaneous and orthotopic tumour xenograft models, and has now entered phase I clinical trials. A further report from Venkatesan *et al.* outlined the development of a related series of 1,3,5-triazine derivatives, targeted with the aim of improving the physicochemical properties of PKI-587 [67]. Incorporation of a 3-oxa-8-azabicyclo[3.2.1]octane group in place of a morpholine resulted in the design of PKI-179, **43**, which displayed potent *in vitro* activity (IC_50_ = 8nM, 74nM, 0.42nM against p110α, p110γ and mTOR respectively). PKI-179 also has high oral bioavailability, and anti-tumour efficacy in the MDA-361 human breast tumour xenograft model. The compound was subsequently advanced into a phase I solid tumour study, though this trial has now been terminated. In a subsequent disclosure, it was revealed that, in *in vitro* studies, a major metabolite of PKI-179, **44**, was generated following incubation in human liver microsomes [68]; Chen and co-workers confirmed that this metabolite displayed comparable *in vitro* potency to that of PKI-179. 

Dehnhardt *et al*. at Wyeth have described the discovery of a series of triazolopyrimidines, which led to the development of PKI-402, **45**, a dual p110α/mTOR inhibitor (respective IC_50_s = 1.4nM and 1.7nM). PKI-402 displayed high anti-proliferative activity in tumour cell lines, induced apoptosis *in vitro*, and conferred potent anti-tumour efficacy in several tumour xenograft models [69]. In a further report, Zhang and co-workers at Wyeth outlined the discovery of a novel class of 5-ureidobenzofuran-3-one indoles also displaying potent p110α/mTOR activity, exemplified by **46**, which displayed very high biochemical activity (IC_50_ = 0.2nM and 0.3nM against p110α and mTOR respectively) with concomitant *in vitro* tumour cell growth inhibition (GI_50_ for inhibition of the growth of PC3 prostate cancer cells = 10nM). Compound **46** also displayed potent anti-tumour efficacy in the MDS-361 breast tumour xenograft model following daily iv dosing [70].

A report from Venkatesan *et al.* outlined the synthesis and characterization of 7H-pyrrolo[2,3-H]quinazolines with PI3K and mTOR activity [71]. The compound with the highest reported mTOR activity was **47** (IC_50_ = 2nM), though the structrually-related **48** displayed potent p110α and p110γ activity (respective IC_50 _values of 22nM and 142nM). Compound **48** also conferred potent *in vitro* tumour growth inhibition (GI_50_ = 48nM, 70nM and 160nM against LNCap, MDA468 and hSMG1 cancer cells respectively).

A series of 4-morpholinopyrrolopyrimidine derivatives was reported by Chen *et al.* to display both p110α and dual p110α/mTOR activity. Compound **49** was found to be a selective and potent p110( inhibitor, with an IC_50_ of 21nM, whilst **50 **had an IC_50_ of 0.9nM and 0.6nM respectively against p110α and mTOR [72].

Chen and colleagues have disclosed the discovery of a series of 2-aryl-7H-pyrrolo[2,3-d]pyrimidin-4-yl)morpholines with activity against class I PI3Ks and mTOR. Compound **51 **showed potent inhibition of p110α and p110γ, with respective IC_50_ values of 0.9nM and 14nM. The tertiary amide derivative, **52**, was reported to display highly potent biochemical inhibition of mTOR inhibition (IC_50 _of 300pM) [73].

Montagne *et al*. at Merck-Serono have reported the synthesis of a library of compounds based upon 2-morpholino-pyrido[3,2-d]pyrimidines which exhibit PI3K activity. One example, **53**, was reported to have an IC_50_ of 8nM [74]. In another report, Cardin and colleagues at Millennium disclosed the production of another targeted library, based upon a thiophene core,****with PI3K activities in the 100nM-5µM IC_50_ range being obtained, including for compound **54 **[75]. An additional library of thiophene derivatives designed by researchers at Millennium, with similar biochemical potencies, was reported by Renou *et al.*, exemplified by **55** [76].

Bo *et al*. at Amgen have disclosed the development of tri-substituted pyridine derivatives displaying dual PI3K/mTOR activity [77], a key example of which was **56**. This compound was reported to have an IC_50_ of 1.3nM and 0.6nM against p110α and mTOR respectively, and displayed high anti-proliferative potency in U87 glioma tumour cells (IC_50_ = 5.3nM). In a further report from Amgen, a series of di-substituted benzimidazole analogues were disclosed by Boezio and colleagues, displaying potent biochemical mTOR activity [78]. It was observed that specific substitution patterns governed selectivity for class I PI3Ks and mTOR; compound **57 **displayed dual mTOR/p110α inhibition (IC_50_ = 0.27nM and 0.65nM respectively), and potent growth inhibition activity in PTEN negative U87 cells (IC_50_ = 5nM).

A new class of imidazo1,2-*a*pyridine derivatives with anti-tumour activity has been disclosed by Bo *et al.* at Amgen [79]. Compound **58 **displayed potent biochemical activity against p110α (K_i_ = 1nM) and mTOR (IC_50_ = 15nM), and potently inhibited U87 glioma tumour cell proliferation (IC_50_ = 7nM).

Rewcastle *et al.* have disclosed a series of morpholino-triazines with specificity for p110α, and which exhibit potent *in vitro* anti-tumour efficacy [80]. A key example is compound **59**, which was seen to be selective over p110β and p110γ (0.1 < IC_50_ < 1 µM), and inhibited NZOV9 cell proliferation with an IC_50_ < 0.1 µM [80].

Heffron *et al*. have disclosed the characterization of GNE-477, **60**. This compound was seen to exhibit dual p110α/mTOR inhibition (IC_50_ = 4 and 21nM, respectively), and displayed potent *in vivo* tumour growth inhibition in the PC3 prostate tumour xenograft model [81].

Cai and colleagues at Curis have reported the generation of a targeted array of small molecules based on the deazapurine, furopyrimidine and thienopyrimidine scaffolds that possess zinc-binding moieties, and which display potent inhibition of p110α, mTOR and histone deacetylase (HDAC) [82]. A representative example is **61**, which showed potent inhibition of all three enzymes (IC_50_ < 100nM), and antiproliferative activities (GI_50_ < 100nM) in a tissue-diverse panel of tumour cells *in vitro*, including the HCT-116, BT-474, SK-MEL-28, and H1993 cancer lines.

Baik and *et al*. at Exelixis have developed a series of pyridopyrimidinones with class I PI3K/mTOR activity [83]. A representative example, **62**, was reported to display p110α, p110β and mTOR inhibition with IC_50 _values of 5.5nM, 52.1nM and 2.6nM respectively, and GI_50 _values of 15.8nM and 97.8nM respectively in PC3 prostate and MCF7 breast cancer cells.

Cheng and colleagues at Pfizer have disclosed the development of PF-04691502, **63**, a dual inhibitor of p110α (IC_50_ = 0.57nM) and mTOR (IC_50_ =16nM), with high *in vivo* efficacy in the p110α mutant SKOV3 ovarian tumour xenograft model. PF-04691502 was subsequently advanced into a phase I, open label, dose escalation study in subjects with solid tumours [84].

Fairhurst and Imblach have reported the discovery of a series of 4,5’-bisthiazoles with potent activity against the class I PI3Ks [85], notably p110α. Representative compounds include **64** (IC_50_, p110α, b, δ, γ = 14nM, 6.51µM, 764nM and 1.28µM respectively) and **65 **(IC_50_, p110α, b, δ, γ = 14nM, 4.94µM, 531nM and 531nM respectively). In a separate report, Caravetti *et al.* outlined the discovery of a related series of compounds again displaying selectivity for p110α, including **66** (IC_50_, p110α, b, δ, γ = 14nM, 4.43µM, 971nM and 680nM respectively), and **67**, which also showed greater potency compared with **66** for p110δ(IC_50_, p110α, β, δ, γ = 8nM, 1.21µM, 77nM and 1.09µM respectively) [86]. 

Kim *et al*. have described the discovery of fluorescent xanthine-based PI3K inhibitors with potent activity in T47D breast cancer cells. The principal biochemical activity of a representative compound disclosed in this report, **68**,****was p110α (IC_50_ = 150nM) [87].

Cheng and colleagues at Pfizer have reported the development of imidazo1,5naphthyridines with p110α and mTOR modulatory activity, and anti-tumour potency. One representative example is compound **69**, which was amongst the most active dual-inhibitors disclosed (Ki, p110α = 167pM; mTOR = 232pM) [88].

Researchers at S*Bio have disclosed the discovery of a class of triazine-based inhibitors with p110α/mTOR dual activity, exemplified by **70** (IC_50_ < 1µM) [89], and have, in a separate report, outlined the development of a series of purine derivatives with similar biochemical potencies, an example of which is **71** [90].

Morales *et al.* at Semafore have developed a class of small molecule anti-tumour agents with class I PI3K and mTOR activity, exemplified by the 7H-thieno[3,2-b]pyran-7-one, **72** [91]. This compound exhibited dual class IA/mTOR activity (IC_50_, p110α, b, δ, γ = 297nM, 378nM, 784nM and 1.57µM respectively; IC_50_, mTOR = 610nM).

Staben *et al.* have reported on the characterization of the p110α inhibitors **73** and **74**, which have respective IC_50_s of 162nM and 6.8nM, and which display potent pharmacodynamic biomarker modulatory activity *in vitro, *notably**effect*s *on**phosphorylation of AKT, PRAS40 and RPS6, in PC3 prostate cancer cells [92].

Finally, Large *et al*. have reported the *in vitro* biochemical and cellular activities of a series of trisubstituted pyrimidines, exemplified by **75** [93]*.* This compound displayed potent activity against p110α (IC_50 _= 62nM), and inhibited the proliferation of IGROV-1 ovarian cancer cells with a GI_50_ of 370nM; the compound also displayed potent down-regulation of phospho-AKT in the same cell line.

### 3.2 Novel Inhibitors of p110β, p110δ and p110γ

Fjellström *et al*. at AstraZeneca have demonstrated that (-)2-[1-(7-methyl-2-(morpholin-4-yl)4-oxo-4*H*-pyrido 1,2-*a*]pyrimidin-9-yl)ethylaminobenzoic acid, **76**, displayed potent inhibition of p110β (IC_50_ = 21nM), with between 4- and 50-fold selectivity over the other PI3K isoforms [94]. In a separate report, Henteman and co-workers at Bayer have reported the discovery of sulfone substituted 2,3-dihydroimidazo[1,2-*c*]quinazoline derivatives, exemplified by **77**, with IC_50_ values against p110γ of less than 100nM [95]. 

Ramsden and co-workers at Cellzome have outlined the production of a targeted array of 2-aminoimidazo[1,2-*b*]pyridazine analogues with PI3K activity, and with potential in the treatment or prophylaxis of immunological, inflammatory, autoimmune or allergic disorders [96]. The most active compound, **78**, displayed high potency against p110γ (IC_50_ < 0.2µM) and 2-50 fold selectivity against the other PI3K isoforms. The same group has also outlined the discovery of 7-substituted aminotriazoles, exemplified by **79 **[97], and urea triazolo[1,5-*a*]pyridine derivatives, exemplified by **80 **[98], both of which display selectivity for p110γ with IC_50 _values of less than 100nM.

Ren and co-workers at Intellikine have reported the synthesis of a library based on a pyrazolopyrimidine core possessing a benzothiazole moiety. These compounds were seen to display activity against the class I PI3Ks and mTOR, with compound **81** having an IC_50_ of less than 50nM against p110δ  and p110γ****and mTOR, through with selectivity over p110β [99]. In a separate report, the same group outlined the synthesis of a series of pyrazolopyrimidines which exhibited activity against one or all PI3K isoforms [100]. Compound **82** was reported to have an IC_50_ of 100nM or less against p110δ****and p110γ whilst having at least 100-fold selectivity against the other two class I isoforms.

Swinnen and co-workers at Merck-Serono have, in two separate reports, outlined the synthesis of libraries of small molecules based upon a [6,5]-heteroaromatic bicyclic core, with selective activity against p110γ, exemplified by **83 **[101] and **84** [102]. In a further disclosure, Pomel and colleagues at Merck-Serono reported the production of a library based on 4-morpholino-pyrido3,2-dpyrimidines, which also displayed preferential activity for p110γ; one example, **85**, was reported to have an IC_50_ of 220nM [103]. 

Researchers at Vertex have reported the development of a series of small molecule heterocycles, exemplified by **86**, which target p110γ [104], and which displayed *in vivo *disease arrest in preclinical models of experimential autoimmune encephalomyclitis (EAE).

Finally, Bruce *et al*. at Novartis have disclosed the development of a new class of small molecules with p110γ activity for use in the treatment of inflammatory and allergic diseases, an example of which is **87** (IC_50_ = 12nM) [105].

## 4 TRANSLATION TO THE CLINIC: MOLECULAR BIOMARKERS

The small molecule inhibitors of the PI3K enzymes described in this review are expected to impact several disease areas, particularly oncology. Key to the translation of targeted therapeutics into the clinic, including PI3K inhibitors described here, is the identification and application of a number of types of biomarker [3]. These range from proof-of-mechanism markers, that can be used to ascertain whether the targeted agent has inhibited the activity of the target and cognate pathway, to prognostic or predictive markers that can be employed to select (or at least enrich) patient populations that are most likely to respond or to determine response to treatment. 

Proof-of-mechanism biomarkers are particularly useful in early clinical studies to confirm target and pathway modulation and to define the pharmacodynamic relationship to dose, toxicity and potential response in the Pharmacological Audit Trail [108]. Biomarkers that have prognostic value, an ability to estimate a given patient’s outcome regardless of the nature of treatment, or predictive value, an ability to estimate the efficacy or the toxicity of an individual patient to a given treatment, allow clinicians to deliver the most appropriate drugs to selected patients and to spare patients unnecessary treatment where they would not benefit from it. It is important to note that nearly half of all new oncology drugs approved by the FDA since the launch of trastuzamab have some form of patient selection biomarker incorporated [109]*.* These biomarkers primarily focus on target biomarkers in tumours that predict patient response, although, more recently, tumour biomarkers of resistance have also been employed, as with mutant KRAS in EGFR-directed therapies. However, although we have made great progress in understanding the complex genetic alterations that underlie human cancer, and there are now several leading examples of molecularly targeted drugs that have exemplified using companion molecular diagnostic assays for patient stratification, it has often proven difficult to identify precisely which molecularly-targeted therapeutics would benefit which particular patients [108]*. *

Significant progress has been made in identifying the most responsive tumour types and the underlying cellular pathways and molecular features determining response to PI3K inhibitors, in addition to pharmacodynamic biomarkers. However, it is clear that the situation is not simple, and that the choice of predictive biomarkers may well depend on the biology and genetics of the tumour type as well as the isoform selectivity profile of the agent concerned. To date several biomarkers potentially predicting tumour sensitivity have been investigated in preclinical and clinical settings, but none has yet been found to have a clearly defined, clinically qualified role for use in patients and thus more research is required. On the other hand, the emerging data show considerable promise and enrichment biomarkers are already in use in early trials of PI3K inhibitors in cancer patients. 

### 4.1 Biomarkers of Mechanism of Action in the Discovery and Development of PI3K Inhibitors

Despite the scientific and technical challenges of identifying a biomarker which specifically and robustly gives a measure of PI3K pathway inhibition, excellent progress has been made and a number of biomarkers are now being used for preclinical and clinical work. The PI3K pathway is a protein kinase cascade activated following the production of PIP3 in the plasma membrane, and this pathway has many nodes and branches, such that there are numerous phospho-proteins that could be potential biomarkers of PI3K inhibition. Quantification of a biomarker close to the point of PI3K inhibition would be the ideal option as this would be the most likely to provide a direct measure or PI3K inhibition. PIP3, the product of the reaction catalyzed by PI3K, would ideally fulfill this requirement for a biomarker of pathway activity. However, while measuring PIP3 using mass spectroscopy, thin layer chromatography or ELISA-based methods with PIP3 detector proteins is feasible *in vitro*, the challenges of measuring PIP3 (both stability and methodology) in samples from patients are generally thought to be very considerable at present. An alternative biomarker to PIP3 could be phosphorylation of a PDK1 substrate, since PDK1 is activated following recruitment to the plasma membrane by production of PIP3. AKT is directly phosphorylated at AKT^THR308^ by PDK1 and this has been widely used as a measure of PI3K activity in *in vitro *tissue culture and *in vivo* tumour xenograft experiments [34]. However, the stability of phosphorylation of the AKT^THR308^ site is generally thought not to be sufficiently robust for use in clinical studies. Other direct protein substrate targets of PDK1, such as SGKs, are yet to be explored as biomarkers of PI3K pathway inhibition. There has been considerable focus on phosphorylation of AKT^SER473^ and some downstream proteins as preclinical and clinical biomarkers of PI3K activity. The downstream markers of activity include PRAS40^THR246^, a substrate of AKT, and RPS6^SER240/244 ^and 4EBP1^THR37/46^. However, none of these biomarkers are perfect as they are not entirely specific for PI3K activation/inhibition because their phosphorylation can be influenced directly or indirectly by mTOR kinase activity. In addition, they may be influenced by inputs from other pathways, for example RPS6 can be phosphorylated by p90^S6K^, a protein kinase regulated by other signalling cascades including MEK/ERK. Nevertheless they can play a useful research role.

A number of studies have used unbiased screening strategies with the aim of identifying better and more specific biomarkers of PI3K inhibition for use in the development of PI3K inhibitors. Andersen and colleagues have employed immunoaffinity precipitation followed by mass spectrometry of protein extracts from cells that were treated with inhibitors of PDK1, AKT or PI3K/mTOR [110]*.* The aim of this study was to find specific biomarkers of PI3K pathway inhibition; it successfully led to the identification and quantification of 375 nonredundant phosphopeptides that were relevant to PI3K pathway signalling, and which contained AKT and PDK1 recognition motifs. Of these, seventy-one phosphopeptides were drug-modulated and 11 were reduced by all three inhibitors examined. An example was phosphorylation of the ribosomal protein RPS6 that was the most strongly inhibited by all 3 inhibitors and phosphorylation of PRAS40^THR246^ which was the most affected following AKT and PI3K/mTOR inhibition. PRAS^THR246^ was validated in lung and breast cancer cell lines and predicted sensitivity to an AKT inhibitor. Importantly, the phospho-PRAS^THR246^ epitope was more stable than the phospho-AKT^SER473^ epitope commonly used for identifying tumours with AKT pathway activation, suggesting that this biomarker might be more suitable for clinical evaluation of PI3K pathway inhibition. In particular it may be ideal for use in immunohistochemistry, which is often applied in clinical studies. 

The value of using ELISA-based methodology to measure quantitatively the phosphorylation of pathway proteins that are both proximal and distal to PI3K has been demonstrated with several inhibitors including GDC-0941, with potency declining at more distal points (34). Interestingly, although inhibition of substrate phosphorylation was valuable as a measure of PI3K target inhibition, the degree of inhibition measured by immune-assay did not predict sensitivity in this study.

An alternative non-biased approach for pharmacodynamic biomarker discovery is to use microarray expression profiling to identify gene signatures specifically associated with PI3K inhibition. Guillard and colleagues profiled gene expression following treatment of human glioma cells with the class I PI3K/mTOR inhibitor PI-103 (**18**) and detected  altered expression of genes encoding regulators of the cell cycle and cholesterol metabolism, together with genes modulated by insulin or IGF1 signalling, rapamycin treatment or nutrient starvation [111]. Expression profiling of *ex vivo* treated peripheral blood mononuclear cells (PBMCs) has also detected a gene signature associated with inhibition of PI3K inhibition; this was validated in microarray expression profiling of mice treated *in vivo *[112]. Further validation of selected cell surface proteins identified from the gene signature determined that the altered expression was specifically induced by PI3K inhibition and not induced by selected cytotoxic agents, MEK inhibitors or the mTORC1 inhibitor rapamycin *in vitro* or *in vivo*.

Some of the biomarkers described herein have been reported as having been examined in early clinical studies of PI3K inhibitors. While it is preferable to look at the effects of PI3K inhibitors on pathway activation in tumours, and this has been done, it is sometimes difficult to access the tumour, or to obtain repeat biopsies. Therefore assessment of PI3K signalling in alternative surrogate normal tissues has also been considered. One option is the hair follicle, which is convenient for repeat sampling and importantly has high PI3K pathway basal activity. For example, in mouse studies the PI3K inhibitor PX-866 decreased phosphorylation AKT^SER473^ in both hair follicles and skin; also NVP-BEZ235 (**10**) has been reported to decrease RPS6^SER240/244 ^and AKT^SER473^ phosphorylation in mouse skin [113,114]. Significantly, early clinical studies of XL765 have reported activity against phosphorylation of PRAS40^THR246^, 4EBP1^THR37/46^, RPS6^SER240/244 ^and AKT^SER473^ in patient hair follicles [115], while another study has reported decreased RPS6^SER240/244 ^in skin samples from patients treated with BMK120 [116]*. *PBMCs and platelet–rich plasma have also been considered as alternate tissues to determine PI3K pathway inhibition. Measurement of AKT^SER473^ phosphorylation in PBMC lysates has proved too variable to be useful; however, analysis of AKT^SER473^ levels in platelet-rich plasma has proved to be a successful alternative, and decreased AKT^SER473^ has been reported following treatment of patients with GDC-0941 and GDC-0980 [117-120]*.* Importantly, the extent of decreased AKT^SER473^ phosphorylation in platelet-rich plasma correlated with the dose of GDC-0941 and was concomitant with decreased RPS6^SER240/244^ phosphorylation in tumour biopsies.

Demonstration of inhibition of PI3K signalling, generally using AKT^SER473^ or RPS6^SER240/244 ^phosphorylation, has also been made in biopsies from solid tumours treated with XL147, GDC-0941, PX-866 and XL765 while a study with the p110δ specific inhibitor CAL-101 reported decreased AKT^THR308^ in isolated lymphocytes from CLL patients [117-119,121-127]*.*

In summarizing this section, various pharmacodynamic and proof-of-mechanism biomarkers have been developed which can be utilised to measure inhibition of the PI3K pathway in tumour biopsies and surrogate normal tissues. Use of these in early clinical trials is providing confidence that the pathway is inhibited by a given drug, and allows optimization of the dose and administration schedule (3). This forms an important part of the Pharmacological Audit Trail that is now important and widely used in the development of molecularly targeted drugs [108].**

### 4.2 Selecting Patients Likely to Respond to PI3K Inhibitors

As PI3K inhibitors progress through the early clinical safety studies and into trials focusing on clinical efficacy, selection of the patient population most likely to benefit from treatment becomes an important consideration [108]*. *A better understanding of drug sensitivity and resistance mechanisms is critical to the successful development and application of targeted cancer agents [128]*. *A good example is the inherent resistance of tumours to anti-EGFR antibody and small molecule therapies resulting from the presence of a KRAS mutation and the sensitivity of patients to the gefitinib and erlotinib EGFR inhibitors in non-small cell lung cancer patients with activating EGFR mutations. We have previously emphasised the importance of identifying predictive biomarkers to select patients that will be responsive or resistant to PI3K or PI3K/mTOR inhibitors [3]. An overview and update is provided here. 

Boyd and colleagues have used reverse phase protein arrays, to profile the phosphorylation status of 100 proteins in a panel of 30 breast cancer cell lines [129]. They found that sensitivity to the PI3K/mTOR inhibitor PI-103 was significantly correlated with elevated phosphorylation at key nodes in the PI3K/AKT/ mTOR pathway, including AKT^THR308^, AKT^SER473^, PRAS^THR246^ and FKHR^T24^, suggesting that high levels of signalling through the pathway may be indicative of pathway addiction and be predictive of response to a targeted PI3K inhibitor. A study by Dan and colleagues came to a similar conclusion in a screen of a panel of 39 cell lines, in which they observed that cancer cell lines with high AKT^SER473^ were more sensitive to a range of PI3K inhibitors from different chemotypes [130]*. *However, there was no correlation observed between the level of AKT phosphorylation and *PIK3CA *mutation status [130,131]. In a separate study, there was no correlation seen between extent of pathway inhibition and sensitivity to PI3K inhibitors such as PI-103 and GDC-0941 [34].

A number of studies with different PI3K inhibitors have demonstrated that tumours with activating *PIK3CA* mutations or loss of PTEN expression are responsive to PI3K inhibition *in vitro* and *in vivo* [34]. Serra and colleagues demonstrated that NVP-BEZ235 had activity in tumours with PI3K activating mutations [114]. Two studies with the early prototype non-specific PI3K inhibitor LY294002 showed that cancer cell lines with PI3K mutations or, conversely, loss of PTEN expression showed increased sensitivity to PI3K inhibition [10,132]. More recent studies with NVP-BEZ235 or GDC-0941 have also shown that tumours with activating *PIK3CA* mutations exhibit increased sensitivity to PI3K inhibition [34,133,134]. These observations would suggest that a patient group with activating *PIK3CA* mutations or loss of PTEN expression would be the most suitable for treatment with PI3K inhibitors. However, the predictive value is not completely clear as, within these studies, there are tumours without *PIK3CA* mutations or loss of PTEN expression that are also sensitive to PI3K inhibition. Moreover, there are a number of *in vitro *or *in vivo* studies of cancer cell line panels that have failed to demonstrate the simple association of *PIK3CA* mutation or loss of PTEN expression with sensitivity to PI3K inhibitors [129,130,135-137]*.* Therefore, at the moment, an informed but pragmatic approach to targeting a patient population with *PIK3CA* mutations or PTEN expression loss with PI3K inhibitors is generally being used – one in which *PIK3CA *mutation and loss of PTEN expression is employed to enrich for patients that will tend to be more likely to respond to PI3K pathway inhibition. At the same time, we must also keep an open mind as it is clear that some tumours without these genetic abnormalities can be equally sensitive to PI3K inhibition, and recognize that the identification and validation of additional predictive biomarkers or signatures will be necessary. This research is ongoing.

A confounding factor in determining the influence of activating mutations of the PI3K pathway on response to PI3K inhibitors may be the presence of other activated oncogenes. Mutations of *KRAS *are frequently co-incident with *PIK3CA* mutations (http://www.sanger.ac.uk/genetics/CGP/Census/). This may be related to the observation that *KRAS* and *PIK3CA* interact and, in mouse tumourigenesis models, *PIK3CA* has been shown to be required for *KRAS*-driven tumourigenesis through direct interaction [138]*. *Similarly, Engleman and colleagues have demonstrated in a mouse model of lung cancer that PI3K signalling is required for KRAS-driven tumourigenesis [139]. In that study, the mouse tumours driven by the *PIK3CA*^H1047^ mutation were responsive to NVP-BEZ235, but not rapamycin. In contrast, tumours driven by mutant *KRAS* were seen to be insensitive to NVP-BEZ235. In a similar vein, Ihle and colleagues noted that mutant *PIK3CA* and loss of PTEN activity were sufficient, but not necessary, as predictors of sensitivity to the anti-tumour activity of the PI3K inhibitor PX-866 *in vivo* in the presence of wild-type RAS, whereas mutant oncogenic RAS was a dominant determinant of resistance, even in tumours with coexisting mutations of *PIK3CA* [135]*.* In support of this, a study of a number of PI3K inhibitors from different chemotypes in a panel of 39 cancer cell lines *in vitro* and 24 *in vivo* tumour**xenografts found a significant association of activating *RAS *or *BRAF* mutations with resistance to inhibition of the PI3K pathway [130]*.* Moreover, an additional study of 84 non-small cell lung cancer cell lines demonstrated reduced sensitivity in those with activating *RAS *mutations [137]*.*

Additional potential factors associated with increased sensitivity to PI3K inhibition have also been identified. Sos and colleagues have shown that the presence of mutated or amplified receptor tyrosine kinase in non-small cell lung cancer cell lines correlated with increased sensitivity and apoptosis following treatment with PI3K inhibitors [137]*.* On the other hand, in the study of Faber and colleagues, inhibition of PI3K/mTOR signalling in non-small cell lung cancers with activating mutations of *EGFR* did not induce apoptosis, in contrast to *HER2*-amplified breast tumours in which sensitivity was seen [140]*. *Several further studies in breast cancer cells have also shown that the presence of amplified *HER2* or the dual presence of *PIK3CA* mutation and *HER2 *amplification increases sensitivity to NVP-BEZ235 and GDC-0941, potentially through an increased cell death response [133,134]*. *In contrast to the *HER2-*amplified breast tumours, inhibition of PI3K/mTOR signalling non-small cell lung cancers with activating mutations of *EGFR* did not induce apoptosis.

Importantly, even if an initial response to targeted therapies is successfully obtained, it is likely that the majority of tumours will at some later point acquire resistance to therapy, and become refractory to treatment [128]*. *This can either be a result of the acquisition of additional mutations or alternatively arise from the outgrowth of a resistant sub-clone already present in the tumour cell population. In the clinic this has been exemplified by tumours that acquire mutations of BCR-ABL, KIT, PDGFR and EGFR during treatment, where the mutated proteins are no longer susceptible to inhibition by the targeted agent, but retain enzymatic activity and the ability to promote cell growth and survival [141,142]*. *As PI3K inhibitors progress through the clinic it is possible that acquired resistance may also become a factor. Zunder and colleagues have addressed this issue using a *S. cerevisiae* screen against a structurally diverse panel of PI3K inhibitors [143]. They identified a potential hotspot for resistance mutations (*PIK3CA*^I800^) and a drug-sensitizing mutation (*PIK3CA*^L814C^). Resistance to small molecule inhibitors of protein kinases can occur as a result of mutations at one particular position in the active site. This mutated residue is known as the gatekeeper because it controls access to a large hydrophobic pocket in which most protein kinase inhibitors bind, and mutations of this type have now been reported for a number of protein kinases. In the case of EGFR, the gatekeeper mutated proteins retain receptor tyrosine kinase activity, but do not bind the EGFR inhibitor [141]. Importantly, the screen of Zunder *et al.* did not reveal resistance mutations at the gatekeeper residue and, in fact, these were unlikely to occur with PI3K inhibitors as introduction of gatekeeper mutations into *PIK3CA* resulted in a loss of enzymatic activity. Thus, the induction of resistance to PI3K inhibitors by this mechanism may be less likely than is the case for many protein kinase inhibitors.

In summary, predictive biomarkers are now emerging that will help us to select patients that are more sensitive to PI3K inhibition. It is already clear that sensitivity and resistance is multifactorial and that the biomarkers already identified, including phosphorylation of PI3K pathway substrates, *PIK3CA *mutation*, *wild type* KRAS* and *BRAF*, loss of PTEN expression, *HER2/ERBB2* amplification, and gene expression signatures, should be seen as useful enrichment biomarkers rather than****truly predictive biomarkers at this time [3,108]*.* There are likely to be confounding molecular factors affecting sensitivity and resistance in cancer cells and in addition the effects of PI3K inhibitors on processes such as angiogenesis and the tumour microenvironment are also likely to be important and contribute to therapeutic activity. The particular tumour type context and the particular isoform selectivity profile of the individual agent may well be important. Continuing research is needed to fully define, scientifically and technically validate, and clinically qualify the predictive biomarkers needed for eventual patient stratification in the event that PI3K inhibitors receive regulatory approval.

## 5 CLINICAL TRIALS IN CANCER

A number of class I and dual class I/mTOR inhibitors have now entered clinical trial [144]. In this section we provide an update on the current status.

### 5.1 Pan-Class I Selective PI3K Inhibitors

Pan-class I selective PI3K inhibitors have been shown to be well-tolerated and, in general, to induce minimal and reversible effects on serum glucose despite the established role of p110α in regulating insulin signalling. Other effects with ATP-competitive PI3K inhibitors include skin toxicity (rash and urticaria). Encouragingly, tumour responses stable disease and other signs of clinical efficacy have been reported in many clinical studies and in a variety of human cancers.

Data on the clinical safety of the peptidic prodrug of LY204002, SF1126, in patients with advanced or metastatic tumours have been reported [145,146]*. *These studies demonstrated good tolerability and activity of the drug administered iv, which led to disease stabilization in patients with refractory tumours, including renal cell carcinoma and chronic lymphocytic leukaemia (CLL). Toxicities that were reported include nausea, vomiting, fatigue, urticaria and rash. No glucose or insulin changes have been reported. Evidence of pathway modulation has been claimed. Dose limiting toxicity has been reported at 1100mg/m^2^.

XL147 was assessed in an open label, phase I dose escalation study that was carried out in patients with advanced solid tumours and lymphoma. Using a standard 3+3 design, patients with solid tumours received once daily XL147 on days 1-21 (21/7) or as a continuous daily dose in 28-day cycles. [121,122]*. *The 3+3 trial design is used for the majority of oncology phase I trials. According to this design, which is simple and straightforward to use, patients are treated in cohorts of 3; then, depending on the number of dose-limiting toxicities seen in the particular patient cohort, decisions are made on which dose to give the next cohort or whether to stop the trial. The maximum tolerated dose was 600 mg in both schedules. The most common drug-related toxicity that was seen was skin rash. Inhibition of PI3K and ERK pathway signalling was demonstrated in solid tumours, and prolonged stable disease has been observed in patients with cancers including non-Hodgkin’s lymphoma and non-small cell lung cancer. Two phase 1 combination studies have been reported with XL147. The combination with the EGFR inhibitor erlotinib was generally well tolerated at doses up to 400 mg XL147/150 mg erlotinib with no major pharmacokinetic interaction and resulted in clinical activity and robust simultaneous inhibition of PI3K and EGFR signalling [147,148]*. *The second combination study with paclitaxel and carboplatin showed that XL147 is well tolerated at doses up to 600 mg in combination with 175 mg/m^2^ and AUC 6 of paclitaxel and carboplatin respectively with no major pharmacokinetic interaction or emerging toxicities. Robust pharmacodynamic activity and tumour regression in heavily pre-treated patients have been observed in this study. Expansion cohorts will include patients with endometrial, ovarian and non-small cell lung cancer. 

Phase I clinical trials are currently being conducted with GDC-0941 [117,118]*. *Initial results have been reported from a phase I study using a 3+3 escalation design with a single dose of GDC-0941 and a 1-week washout, followed by GDC-0941 QD administered on a 3-week on, 1-week off schedule. A dose-proportional increase in drug exposure was observed from 15 to 450mg. Target modulation was reported with inhibition of AKT^SER473^ phosphorylation in platelet-rich plasma at doses above 80mg and a decrease in RPS6 immunostaining in tumours. In addition, objective decreases in metabolic activity as measured by positron emission tomography of fluorodeoxyglucose (^I8^FDG-PET) have been observed in patients’ tumours at doses above 80mg. GDC-0941 was generally well tolerated and exhibited signs of anti-tumour activity in a variety of cancers including breast, ovarian cancer, gastro-intestinal stromal tumour (GIST) and melanoma patients. Toxicities include fatigue, nausea, diarrhea and rash. Transient hyperglyceamia has been described. GDC-0941 is being evaluated in non-small cell lung cancer in combination with paclitaxel and carboplatin with or without bevacizumab. So far, these combinations appear to be well tolerated and no sign of pharmacokinetic interaction have been observed. Dose escalation is ongoing and clinical activity has been recorded [119]*. *A phase II study in breast cancer is recruiting 

In an initial phase 1 dose-escalation study evaluating an intermittent dosing schedule, PX-866 was well tolerated with diarrhoea and nausea observed as main toxicities. PX-866 was rapidly converted to an active metabolite (17-OH PX-866) which demonstrated improved potency relative to parent compound in kinase and cellular assays [123]. PX-866 was further evaluated using a continuous dosing schedule and has been well tolerated at 8 mg per day and associated with better disease control in heavily pre-treated patients than intermittent dosing. Clinical responses have been observed in pancreatic islet cell, colorectal, and prostate cancer. Predictive biomarkers are being explored. 

Patients were treated at 6 doses of BMK30 ranging from 12.5 mg to 150 mg [116]*.* The maximum tolerated dose was 100 mg. Treatment-related adverse events included rash, hyperglycaemia, diarrhoea, nausea, anorexia, pruritus, fatigue, mood alteration, malaise, vomiting, and mucositis. Preliminary pharmacokinetic analysis showed rapid absorption and low clearance from plasma leading to steady-state drug exposure estimated to be potentially efficacious based on preclinical data. Downregulation of pS6 in skin was seen in all patients at 100/150 mg. At 100 mg, 8 of 10 evaluable patients showed metabolic partial response by FDG-PET. Clinical responses were observed in triple negative breast cancer, colorectal cancer, angiosarcoma and lung cancer.

### 5.2 Dual Pan-Class I PI3K/mTOR Inhibitors

The safety profile and tolerability of the dual pan-PI3K/mTOR inhibitors generally appears to be similar to that of the pan-inhibitors. Several organizations are developing candidates with both profiles and it is currently unclear what the ideal PI3K family isoform selectivity profile or profiles in the clinic will be. Signs of clinical activity are also encouraging for the development of these agents.

The first reports from clinical trials conducted in patients with solid tumours showed promising drug safety and tolerability for NVP-BEZ235 with signs of clinical activity in patients with tumours bearing PI3K pathway alterations [149]*. *Toxicities that were reported included nausea, vomiting, diarrhea, fatigue/asthenia, anemia, and anorexia; these effects were mild or moderate, manageable, and reversible upon treatment discontinuation. AUC and C_max_ were found to increase non-proportionally with dose and were variable within and among patients. NVP-BEZ235 exhibited dose- and day-dependent PI3K inhibition as measured by elevation of plasma C-peptide levels. 2 partial responses (1 Cowden syndrome patient, 1 breast cancer patient) and 16 measurable responses were observed. 14 of 51 evaluable patients had stable disease for ≥4 months; tumours from 6 of these 14 patients carried dysregulation of the PI3K pathway. Four of the 14 (29%) patients with stable disease for ≥4 months had breast cancer. 18 of 35 evaluable patients had detectable decreases of ^18^FDG uptake. An improved formulation of the compound will be used in future studies. 

XL765 was administered twice daily (BID) or daily (QD) for 28-day cycles with a standard 3+3 dose escalation design in patients with solid tumours and lymphoma. The most common related adverse events (> 10% of patients) were observed to be nausea, diarrhoea, anorexia, elevated liver enzymes, skin disorders, and vomiting. Exposure was found to be increased with increasing doses on BID and QD schedules. Robust pharmacodynamic modulation of PI3K and ERK pathway signalling was evident both in tumours and surrogate tissues following dosing of XL765. For example, decreases in phosphorylation of AKT^THR308^ (57-76%) or of 4EBP1 (62-80%), as well as ERK (53-80%), were observed in paired biopsies of various solid tumours from patients receiving 50 mg BID. Eleven patients have been reported to be on study for ≥ 16 weeks and seven patients on treatment for ≥ 24 weeks.****The maximum tolerated dose for single-agent XL765 is reported as 50 mg BID. XL765 exhibited potent pharmacodynamic activity in solid tumours and surrogate tissues at generally well tolerated doses [126]. XL765 in combination with the DNA methylating agent temozolomide is well tolerated at doses up to 40 mg QD. There was no apparent pharmacokinetic interaction between XL765 and temozolamide. Maximum tolerated dose determinations for QD and BID schedules are ongoing. Signs of disease stabilisation have been observed. XL765 in combination with erlotinib is also generally well tolerated at daily doses up to 50 mg XL765/100 mg erlotinib with no apparent pharmacokinetic interaction, and results in robust inhibition of PI3K and EGFR signalling in skin and tumour tissue. The maximum tolerated dose for the combination has not yet been determined [124,125].

The phase I dose escalation study of GDC-0980 was carried out in patients with solid tumours or non-Hodgkin's lymphoma and used a 3+3 design [120].**GDC-0980 was given on day 1, followed by 1week washout to investigate single-dose pharmacokinetic and pharmacodynamic biomarkers. The most frequently reported adverse events were nausea (25%), fatigue (50%), diarrhoea (42%), and flatulence (25%). GDC-0980 was found to be generally well tolerated up to 16 mg administered orally QD with potential signs of anti-tumour activity. Preliminary pharmacokinetic data suggest dose-proportional increases in Cmax and AUC. Initial pharmacodynamic biomarker data showed >50% inhibition of phosphorylated AKT^SER473^ levels assayed in platelet-rich plasma after a single dose of 8 mg and higher of GDC-0980. Of potential interest, evidence of anti-tumour activity was observed in a mesothelioma patient previously treated with radiation and cisplatin. The recommended dose is yet to be established 

Two candidates from Pfizer are currently being developed, one for i.v. administration (PF-04691502), and one for oral dosing PF-05212384**[150,151]. Both compounds are dual PI3K/mTOR inhibitors and show acceptable pharmacokinetic profiles after 3 dose escalations. So far no clinical activity has been reported. Pharmacodynamic biomarker assessment is being performed by measurement of glucose and insulin levels in blood. Nausea, fatigue, headache and hyperglycaemia have been the most frequently reported treatment-related adverse events so far. Dose escalation is ongoing for both compounds 

### 5.3 p110δ-Selective PI3K Inhibitors

The potent p110δ-specific inhibitor CAL-101 exhibits 40- to 300-fold selectivity for that particular isoform, as compared to other PI3K enzymes and is undergoing Phase I clinical evaluation in relapsed or refractory haematological malignancies (CLL, acute myeloid leukaemia, multiple myeloma and non-Hodgkin’s lymphoma) [127]*. *The first interim reports from phase I trials with CAL-101 show promising drug activity and a lack of severe toxicity in haematological cancer patients. Plasma exposure was shown to increase with dose. AKT^THR308^ as a marker of PI3K activation was measured in cells from a subset of chronic lymphocytic leukaemia patients with circulating lymphocytes and was observed to be reduced by >90% following dosing, demonstrating target inhibition. 

## 6 SUMMARY AND FUTURE PERSPECTIVE

As mentioned earlier in this review, the progression of PI3K inhibitors over the last twenty years or so has been remarkable. There are a number of interesting and important features that can be highlighted.

First is the evolution from chemical tool compounds, like LY294002 **1**, Wortmannin **2** and PI-103 **18** [152], to drugs that are now beginning to show pharmacodynamic evidence of target modulation and clear signs of therapeutic benefit to cancer patients [3,153].

Next to highlight is the impact of the crystal structures of p110 catalytic domains, facilitating the interpretation of isoform selectivity profiles and the prospective design of desired profiles [3,154-156]*. *In the landmark study by Knight *et al. *[106],**common selectivity combinations were identified, as with agents that exhibit preferences for p110α/p110γ and p110β/δ (termed ‘pharmalogs’). Inhibitors of p110α often also inhibit the class IV isoforms DNA-PK and mTOR, as with PI-103, but it has been possible to remove the class IV inhibition from class I-selective inhibitors as in case of GDC-0941 **3** compared to PI-103 [34]. The desirability of p110α/pan-class I isoform selectivity for cancer therapy is still being debated [3,34,107]*. *It is now clear that highly selective inhibitors of p110δ can be produced and that p110γ/δ inhibitors can also be obtained, for potential use in inflammation. Many variations on these core patterns exist. Although mouse models will help, it is likely that the preferred isoform selectivity profiles for medical use, as distinct from chemical tools, will only emerge following detailed clinical evaluation of multiple agents. For future drug design, the SAR rules for achieving selectivity are progressively being defined, facilitated by the increasing availability of crystal structures. In addition to the various isoform-targeted inhibitors developed to date, there is also significant emerging potential for p110β and dual p110β/δ inhibitors for the treatment of immune-inflammatory diseases and cancer [19, 12, 157-160] and also of p110γ inhibitors in the latter therapeutic area [161].

Also important has been the use of proof of mechanism pharmacodynamic biomarkers to demonstrate target and pathway modulation in both the preclinical discovery phase and the early clinical development of PI3K inhibitors. This is critical in the implementation of the Pharmacological Audit Trail [108]*, *enabling rational optimization of dose and schedule of administration as well as go/no go decision-making. In addition, progress has also been made on the identification of potential predictive biomarkers for the identification of patients that are most likely to respond to PI3K inhibitors. These include *PIK3CA* mutation, PTEN expression loss, *HER2/ERBB2 *amplification/overexpression, wild type *KRAS* and gene expression signatures.

Finally to be highlighted is the emerging picture from the clinic of PI3K inhibitors as generally well tolerated agents that are already beginning to show evidence of single agent therapeutic activity in early clinical trials in cancer patients. Concerns about potential effects on glucose metabolism appear to have been alleviated, with only mild effects being seen, at least with the doses and schedules used to date.

What then are the key issues facing the preclinical discovery and clinical development of class I PI3K and class I/class IV inhibitors for cancer treatment?

Identifying optimal isoform selectivity profiles has already been discussed and is ongoing. Related to this point, more work needs to be done to identify the best predictive markers of sensitivity for drugs with different selectivity profiles. In addition, further research on biomarkers of resistance, both intrinsic and acquired, is also essential. At the moment the available biomarkers are probably best described as enrichment biomarkers – for use in enriching early clinical trials for patients with malignancies with molecular characteristics (PI3K pathway addiction) that make them more likely to respond. Much more work needs to be done to validate and clinically qualify biomarkers that may be truly predictive. It needs to be remembered that, especially since PI3K inhibitors can have effects on tumour angiogenesis and tumour microenvironmental interactions, there may not be a single biomarker of sensitivity but rather a group of these or a predictive molecular signature. Studies in preclinical systems, including large molecularly characterised cancer cell panels and human tumour xenografts, together with genetically engineered mouse models, will be useful for this. However, it is likely that many of the answers will be worked out by molecular profiling, including cancer genome sequencing, of clinical tumour material and the correlation of such data with therapeutic response and outcome.

Many PI3K inhibitors are now progressing through phase II single agent efficacy studies and the results are eagerly awaited by the oncology community. Combination studies are also underway. Because of the number of potential combinations, it may take some time to identify optimal combinations and both preclinical and clinical studies will be important for this. Some rationally based combinations, for example with MEK inhibitors, have obvious mechanistic appeal and these are being prioritized [3].

In addition to cancer indications, there is exciting potential for PI3K inhibitors in other therapeutic contexts, particularly immune inflammation and cardiovascular disease. 

It has been a fascinating journey so far with PI3K inhibitors. With the range of agents now coming through that have distinct and attractive profiles, in the next few months and years there should increasing opportunities to reveal further evidence of clinical utility. 

## Figures and Tables

**Fig. (1):**
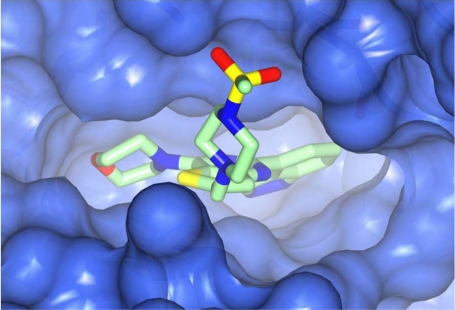
X-ray co-crystal structure of the pan-class I selective PI3K inhibitor GDC-0941 (3) (PDB Code 3dbs). For more details see text and references [3, 33, 34].

**Table 1:** 

ENTRY	STRUCTURE	COMMENTS	REF
1	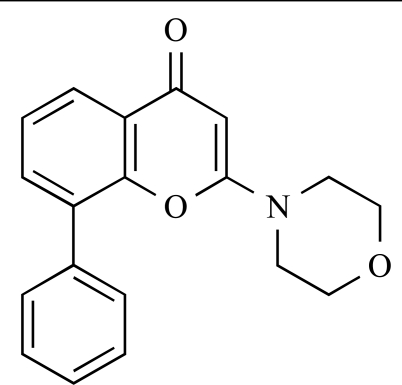	LY294002: IC_50_ of 0.55µM, 11µM, 1.6µM and 12µM against p110α, β, δ and γ respectively	[28]
2	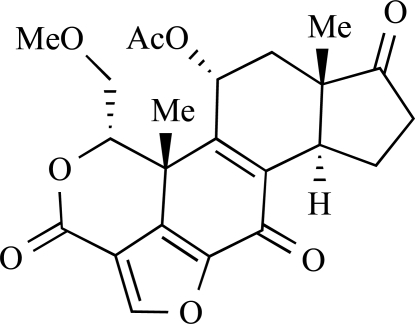	Wortmannin: IC_50_ of 4, 1nM, 4 and 9nM against p110α, β, δ and γ respectively	[29]
3	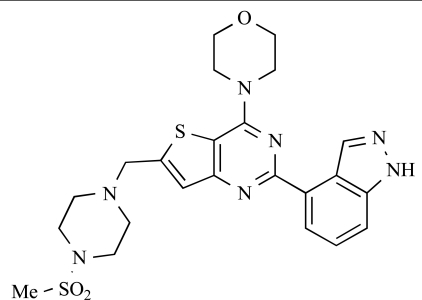	GDC-0941: IC_50_ of 3, 33, 3 and 75nM against p110α, β, δ and γ respectively	[33, 34]
4	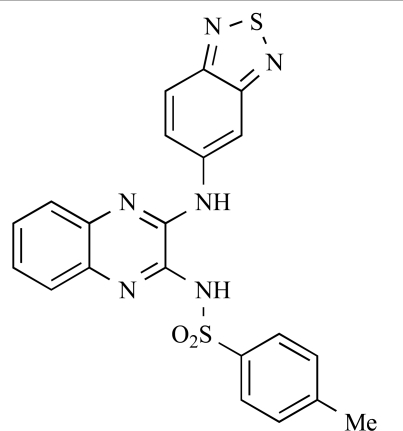	XL147: IC_50_ p110α, β, d, γ = 39, 383, 36, and 23nM respectively	[35]
5	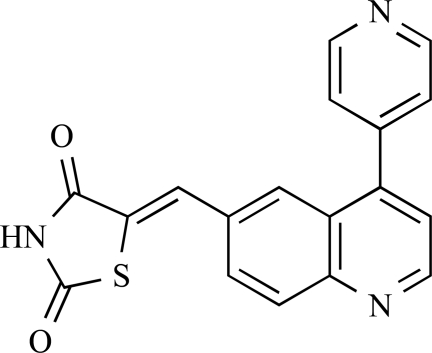	GSK1059615: IC_50_ p110α, β, d, g and mTOR = 0.4, 0.6, 2, 5 and 12nM respectively	[36]
6	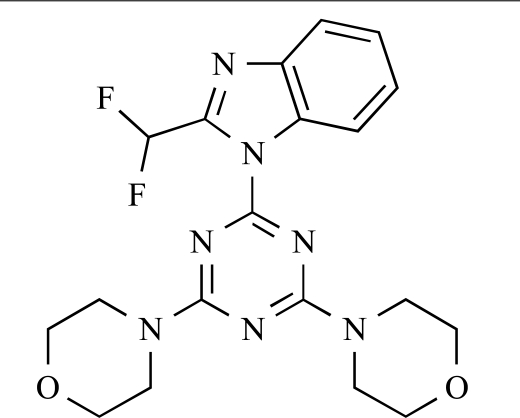	ZSTK474: IC_50_ p110α, β, d, γ = 16, 44, 5, and 49nM respectively	[37]
7	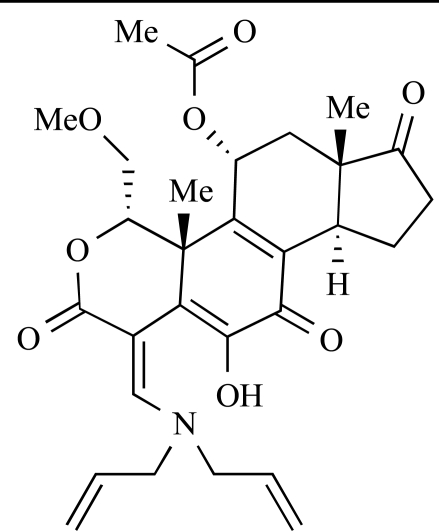	PX-866: IC_50_ p110α, d and γ = 6nM, 3nM and 9nM respectively; p110β > 300μM	[29]
8	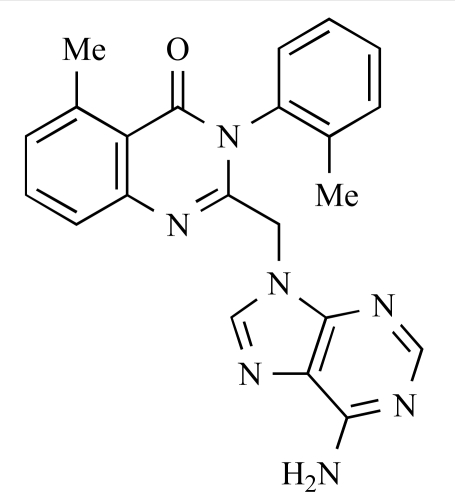	CAL-101: IC_50_ p110α, β, δ and γ = >100μM, 1.82μM, 70nM and 1.24μM respectively	[38]
9	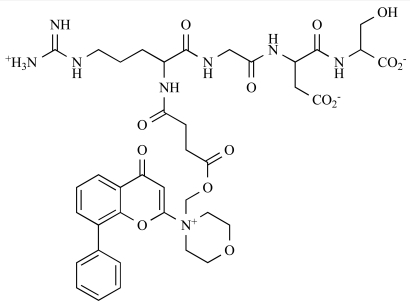	SF1126: IC_50_ p110α, β, δ and γ = 356, 736, 3225, 1774nM respectively; IC_50_ mTOR, DNA-PK = 1060 and 357nM respectively	[39]
10	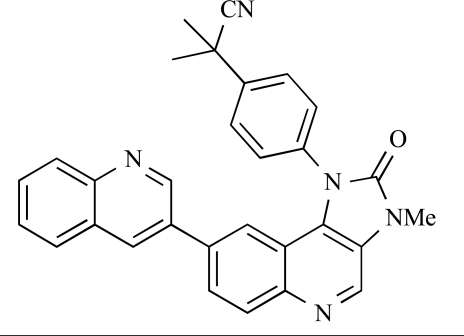	NVP-BEZ235: IC_50_ p110α, β, d, g and mTOR = 4, 75, 7, 5, 21nM respectively	[40]
11	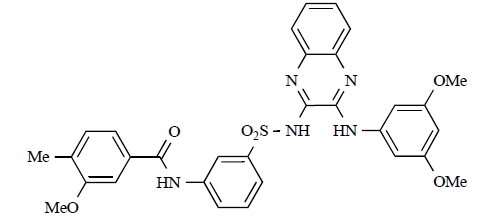	XL765: IC_50_ p110α, β, d, g and mTOR= 39, 113, 43, 9 and 157nM respectively	[41]
12	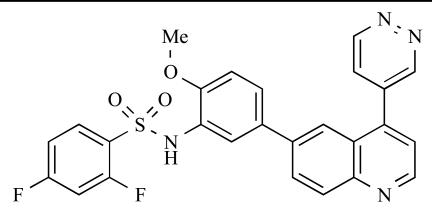	GSK2126458: Ki p110α, β, d, g and mTORC1 = 19, 130, 24, 60 and 180pM respectively	[36]
13	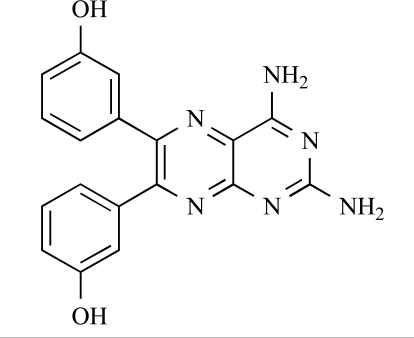	TG100115: IC_50 _p110α, β, δ and γ = 1.3μM, 1.2μM, 235nM and 83nM respectively	[29]
14	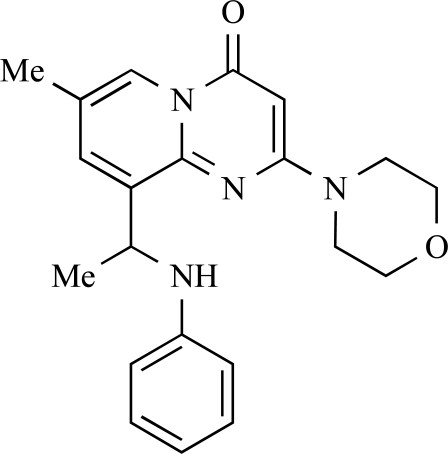	TGX 221: IC_50_ p110β = 5nM; p110α = 5μM, p110δ = 100nM, p110γ > 10μM	[42]
15	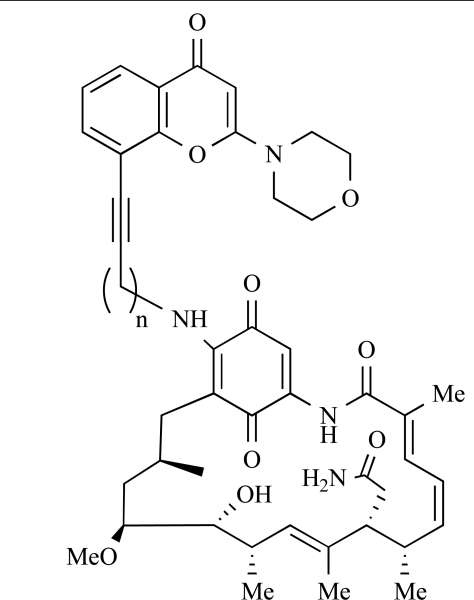	Heterodimer of LY294002 and geldanamycin exhibits activity against PI3K and Hsp90	[43]
16	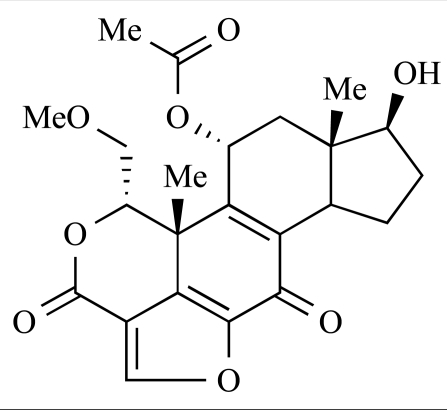	17-Hydroxywortmannin: IC_50 _p110α = 2.7nM	[44]
17	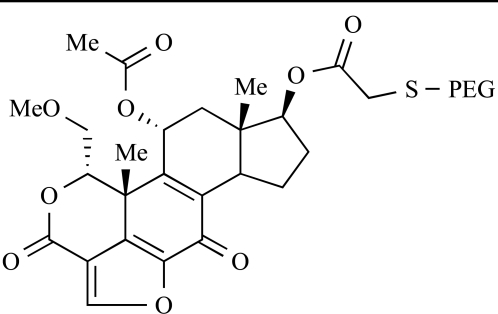	PWT-458, a pegylated derivative of 17-hydroxywortmannin with improved tolerability profile compared with 16	[45]
18	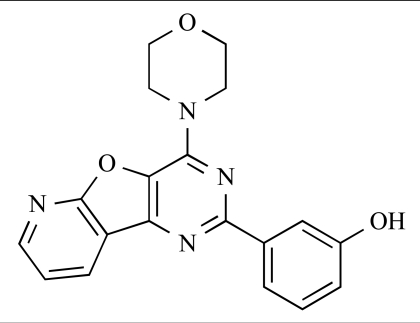	PI-103: IC_50_, p110α = 3.6nM, p110β = 3nM, mTORC1 = 20nM, mTORC2 = 80nM	[46, 4]
19	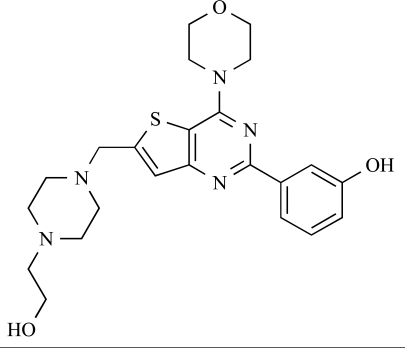	IC_50_, p110α = 7nM, p110γ = 670nM	[34]
20	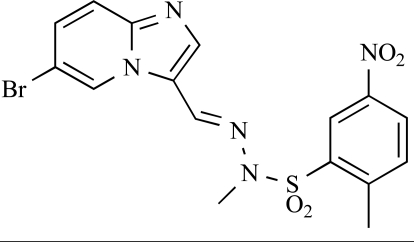	IC50, p110α = 3nM, p110β = 170nM, p110γ = 230nM	[47]
21	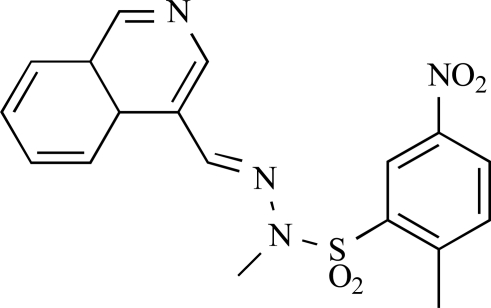	IC50, p110α = 800nM, p110β >10μM	[48]
22	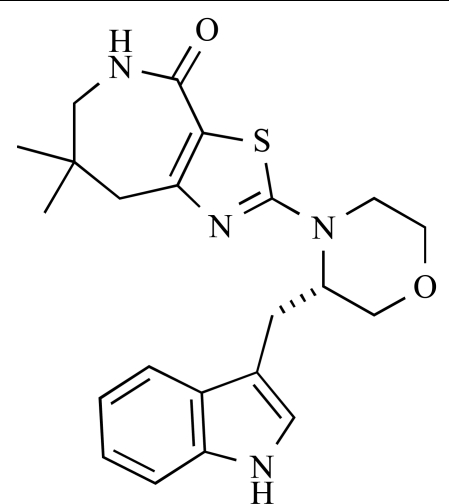	IC_50_ = 59nM, 1.006 µM, 18nM, 31nM for p110 α, p110β, p110δ and p110γ respectively	[49]
23	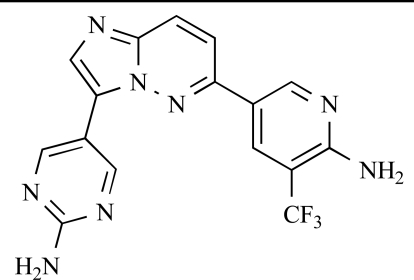	IC_50_ p110α = 15nM, p110β =4nM, p110δ = 9nM, p110γ = 737nM	[50]
24	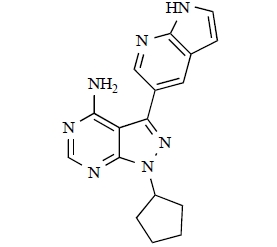	IC_50_, p110α, DNA-PK and mTOR of 52nM, 60nM and 10nM respectively; IC_50 _ p110β and p110γ = 1.4µM and 1.1µM respectively	[51]
25	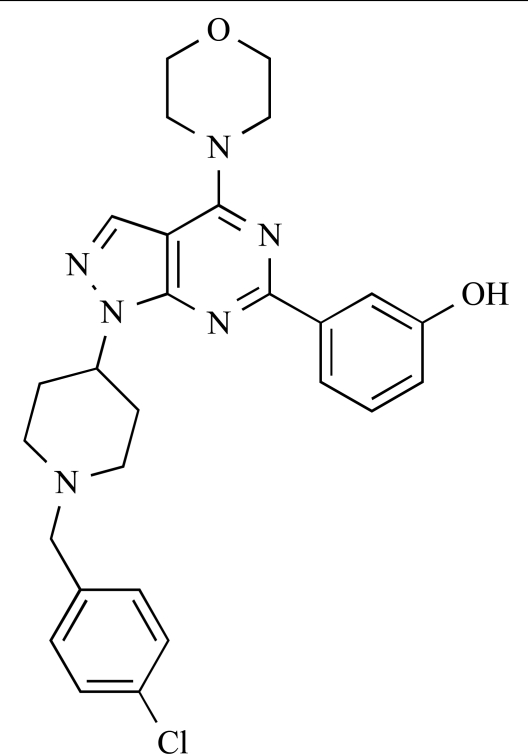	IC_50_, p110α = 11nM, mTOR = 17.5nM; GI_50_, LNCaP = 450nM, MDA468 = 1.7µM	[52]
26	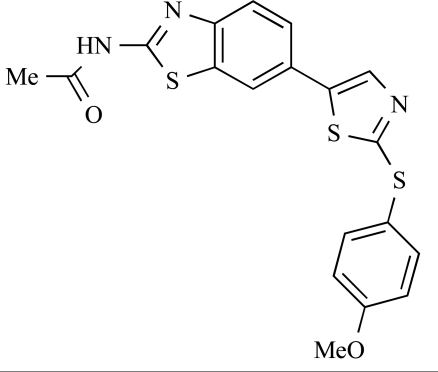	IC_50_, p110α = 29nM, p110β = 5.2µM	[53]
27	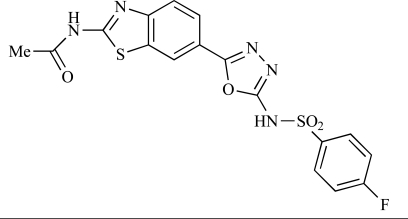	IC_50_, p110α = 3nM, p110β = 3.8nM	[53]
28	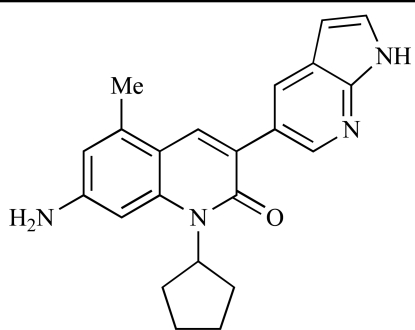	IC_50_, p110α = 500pM	[54]
29	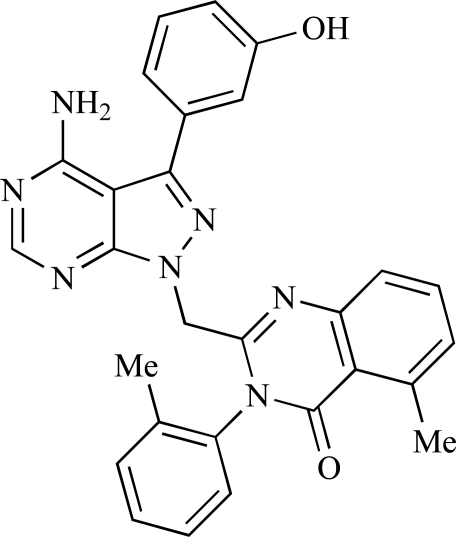	IC_50_, p110δ = 3nM; >60-fold selectivity over p110γ; >200-fold specificity over p110α, p110β, DNA-PK and mTOR.	[51]
30	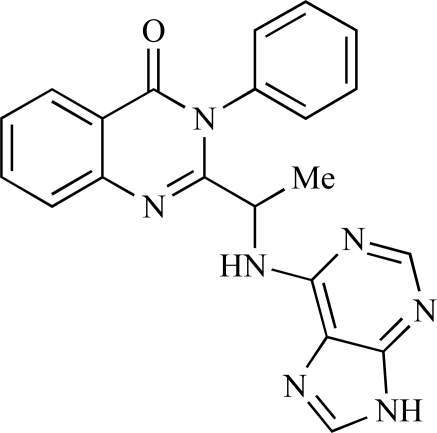	Ki, p110δ = 5nM	[55]
31	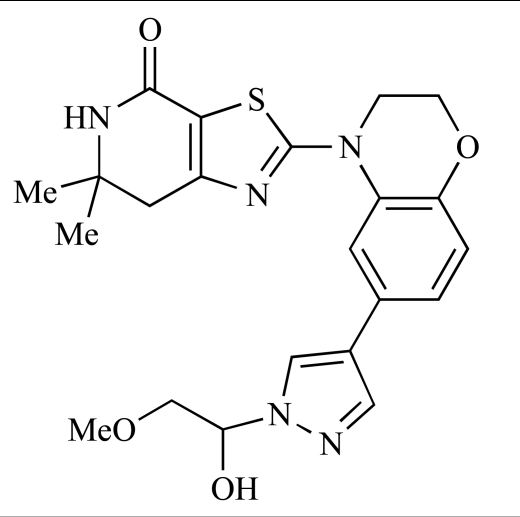	IC_50_, p110δ = 14nM; IC_50_, p110γ = 52nM	[56]
32	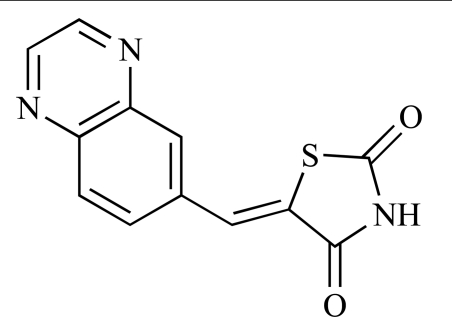	AS-605340: IC_50_, p110γ = 8nM	[57]
33	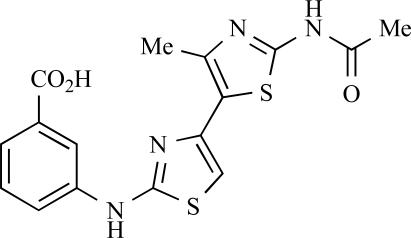	IC_50_, p110γ = 10nM IC_50_, p-AKT = 3.18µM	[58]
34	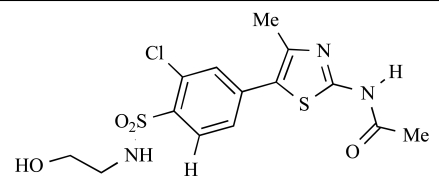	IC_50_, p110γ = 2nM	[59]
35	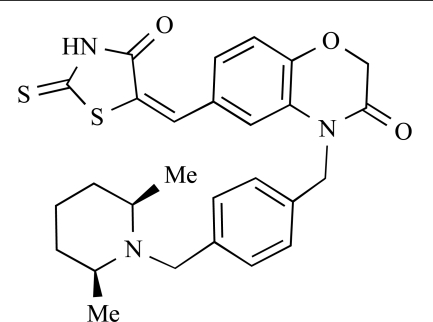	IC_50_, p110γ = 3nM	[60]
36	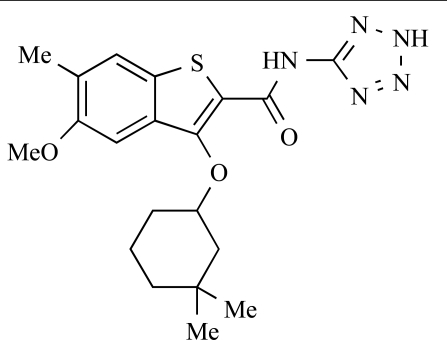	IC_50_, p110γ = 3nM	[61]

**Table 2:** 

ENTRY	STRUCTURE	REF
37	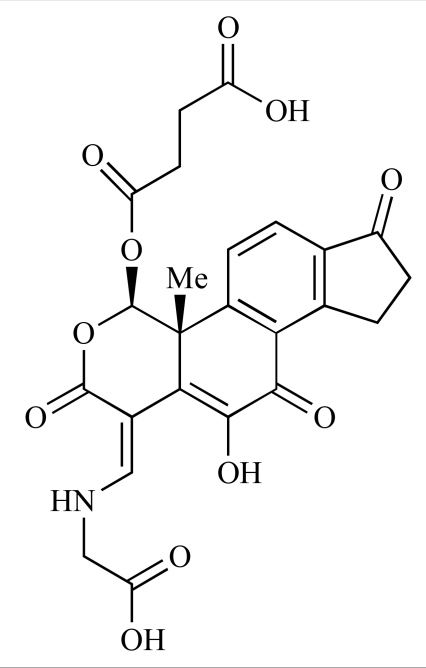	[62]
38	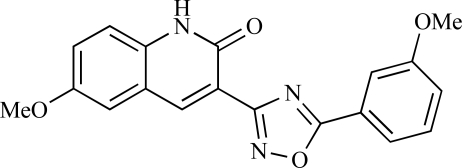	[63]
39	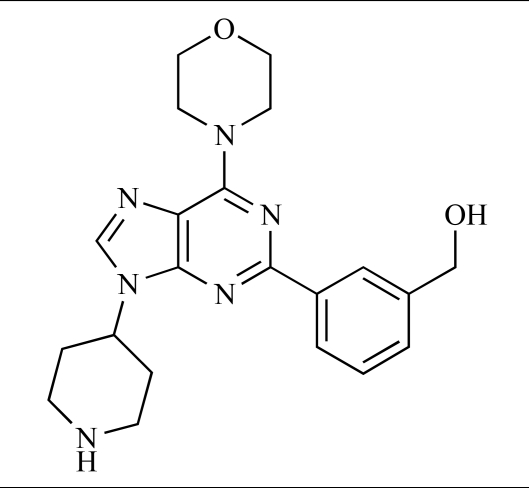	[64]
40	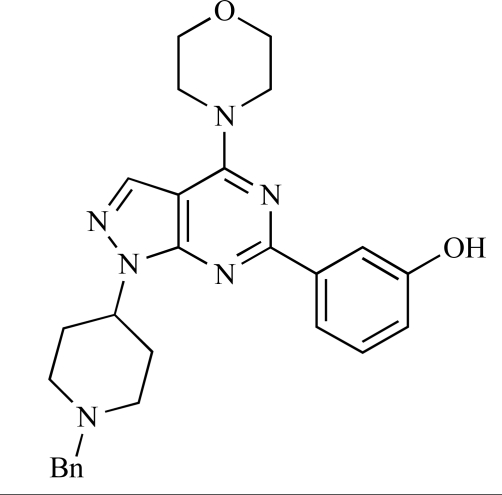	[64]
41	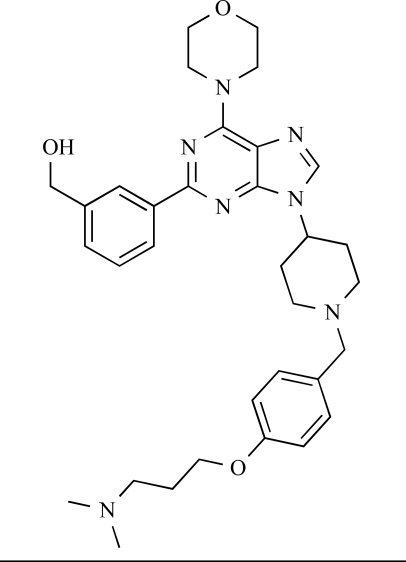	[65]
42	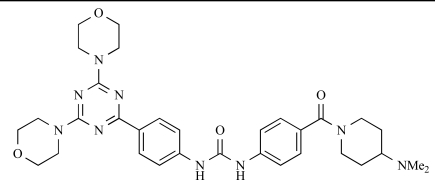	[66]
43	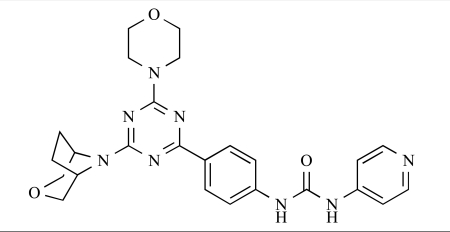	[67]
44	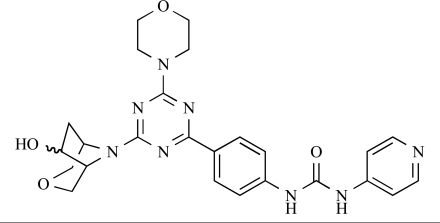	[68]
45	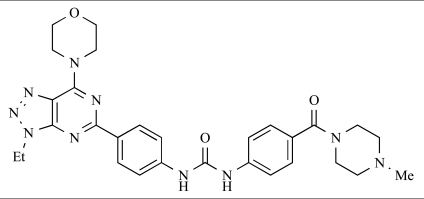	[69]
46	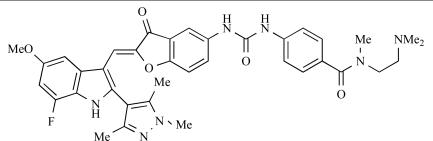	[70]
47	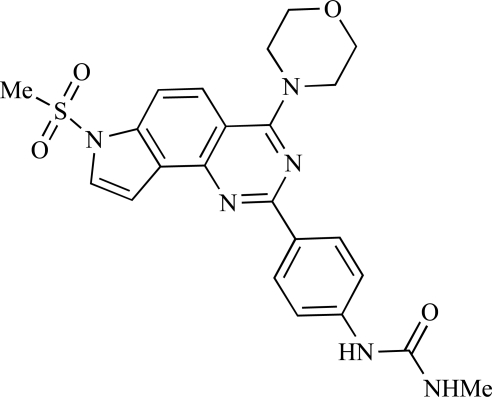	[71]
48	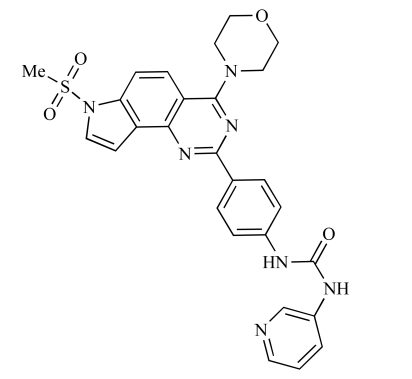	[71]
49	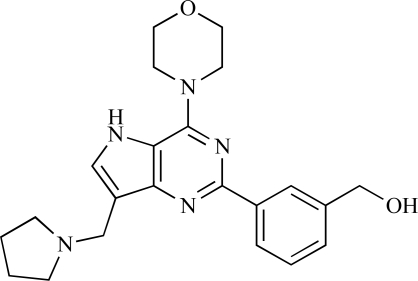	[72]
50	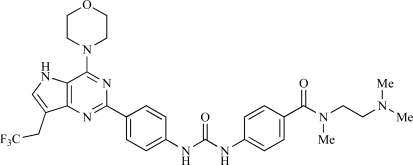	[72]
51	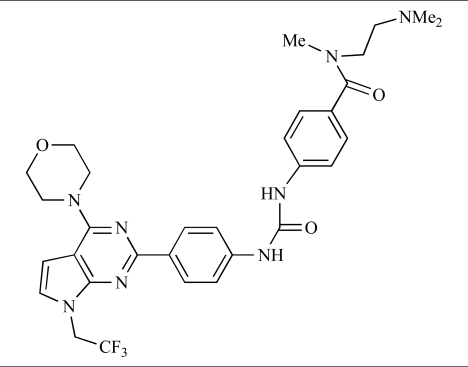	[73]
52	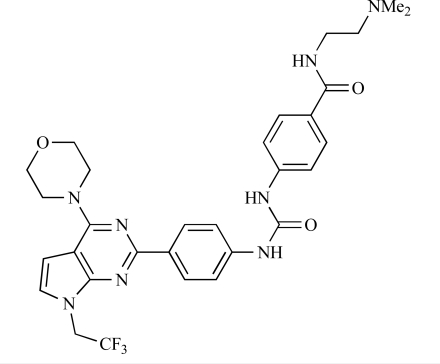	[73]
53	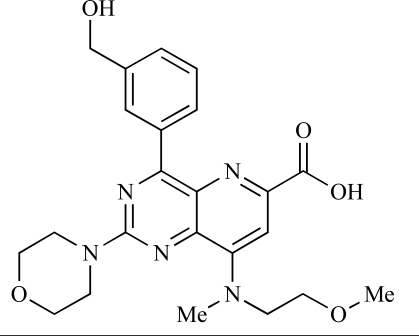	[74]
54	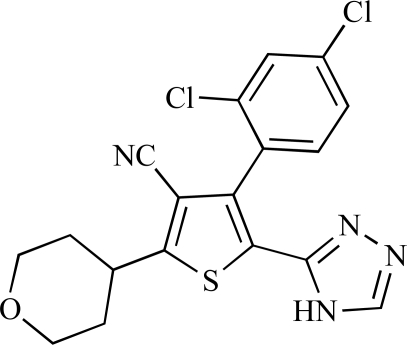	[75]
55	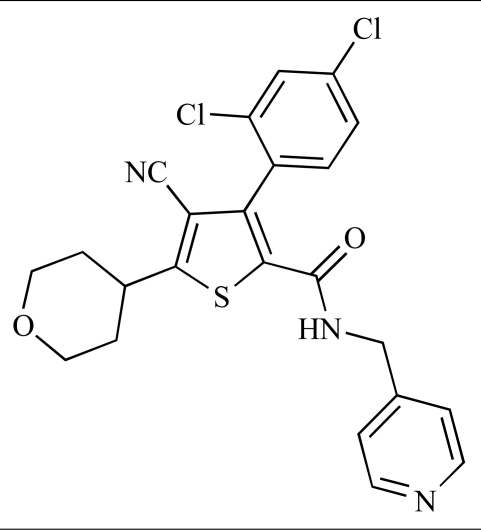	[76]
56	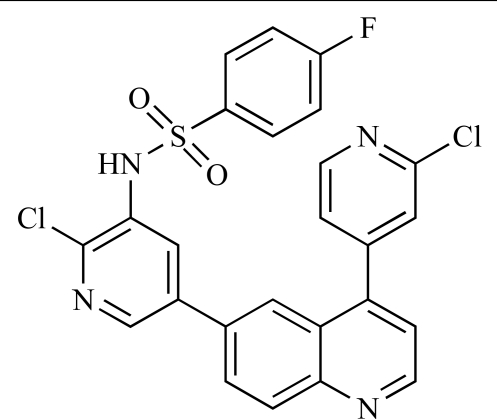	[77]
57	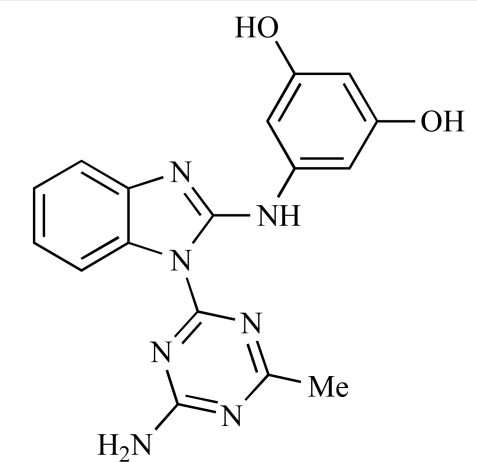	[78]
58	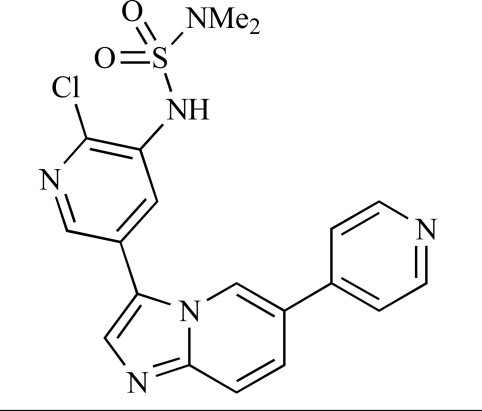	[79]
59	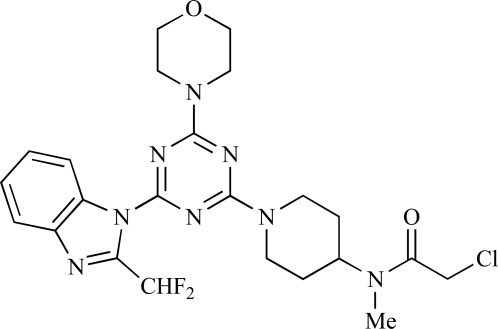	[80]
60	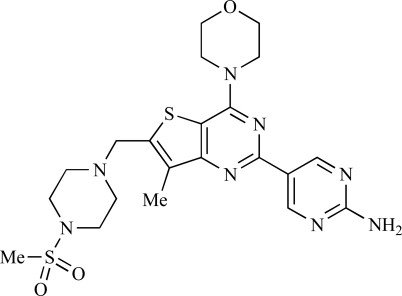	[81]
61	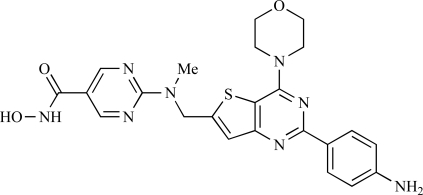	[82]
62	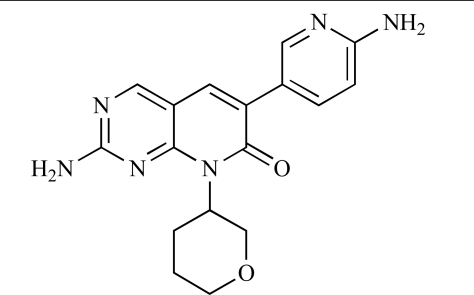	[83]
63	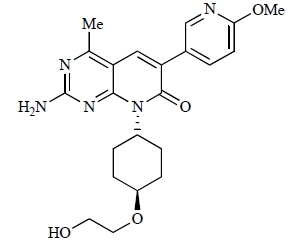	[84]
64	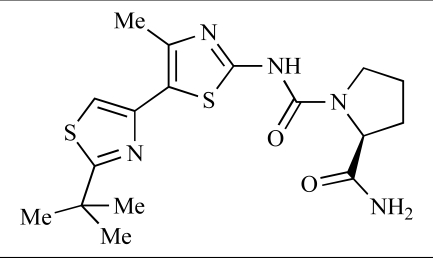	[85]
65	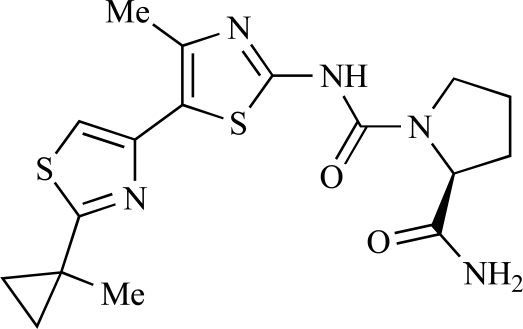	[85]
66	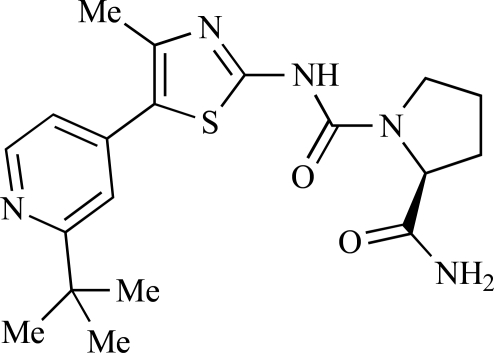	[86]
67	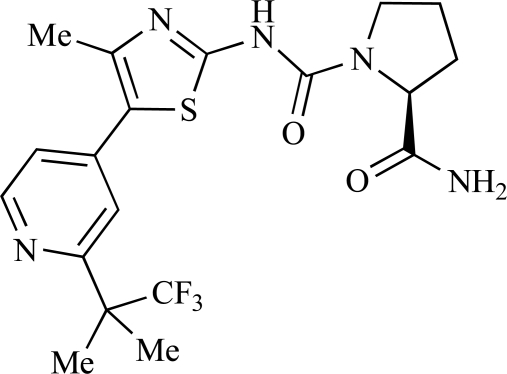	[86]
68	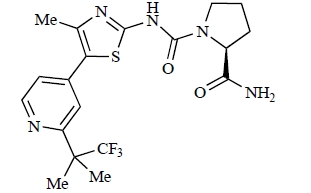	[87]
69	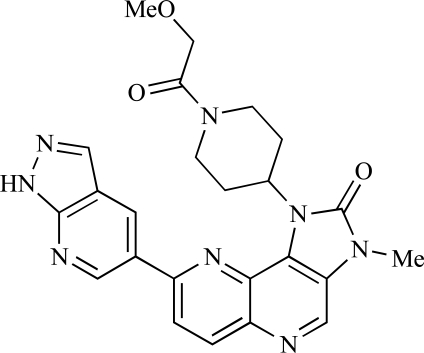	[88]
70	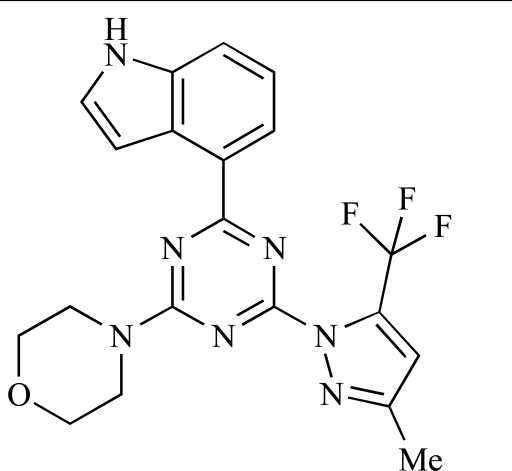	[89]
71	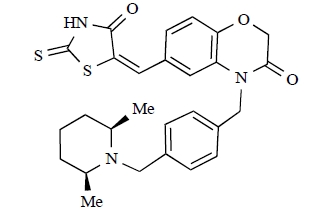	[90]
72	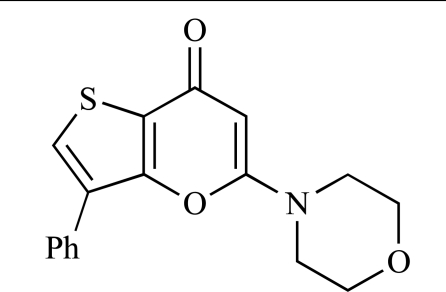	[91]
73	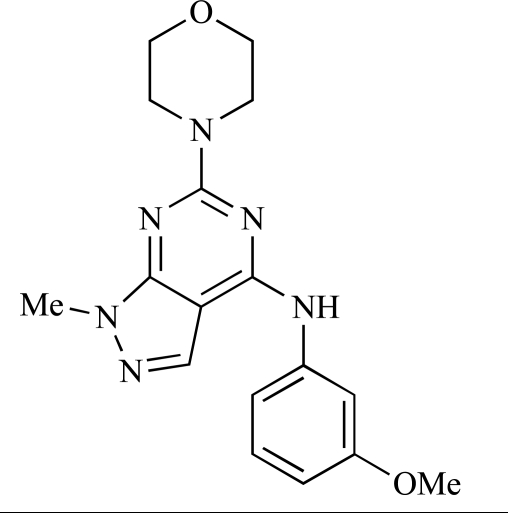	[92]
74	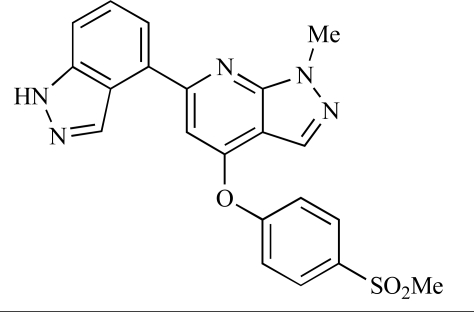	[92]
75	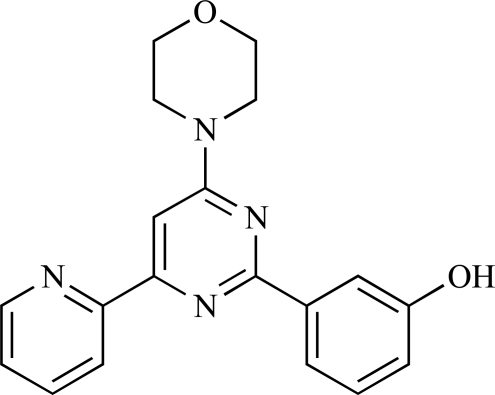	[93]
76	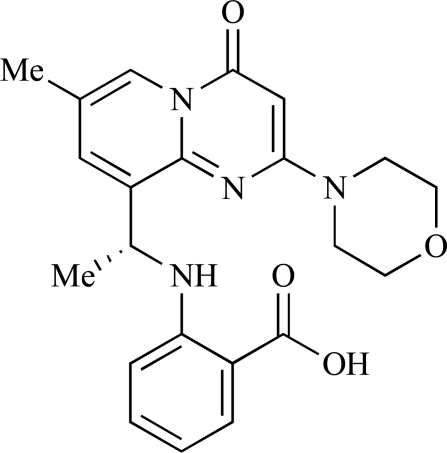	[94]
77	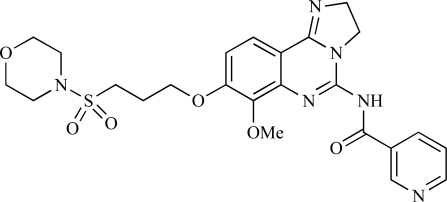	[95]
78	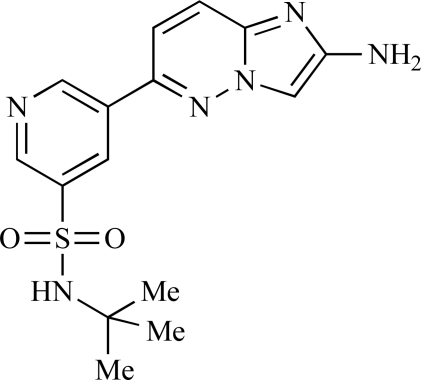	[96]
79	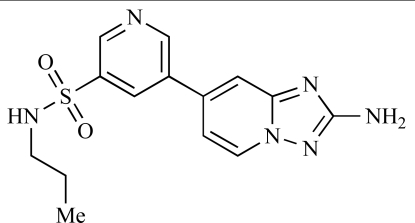	[97]
80	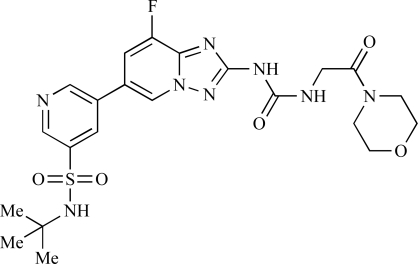	[98]
81	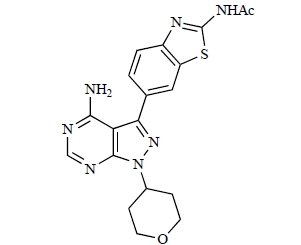	[99]
82	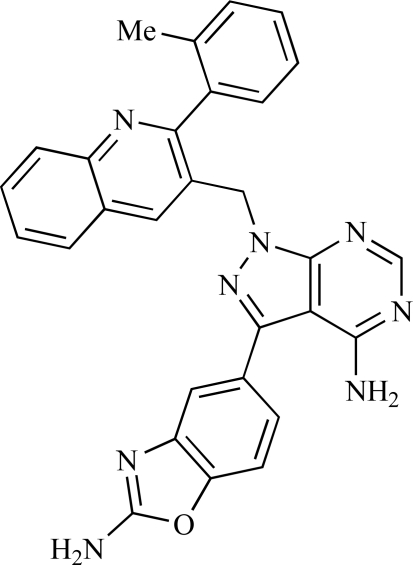	[100]
83	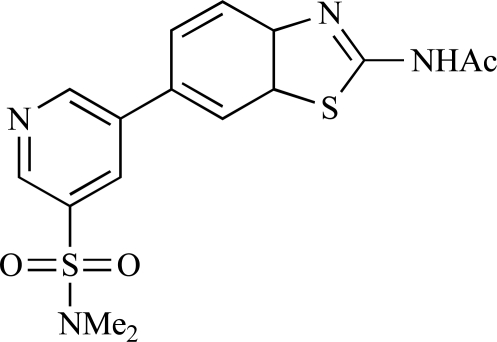	[101]
84	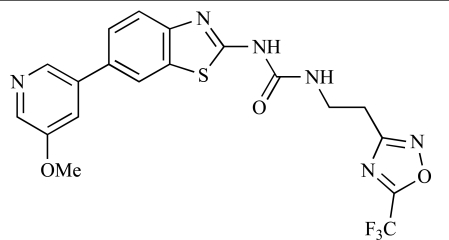	[102]
85	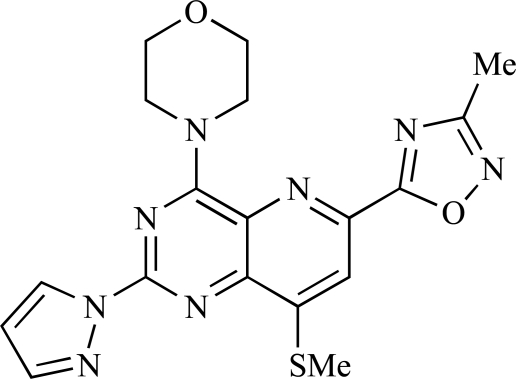	[103]
86	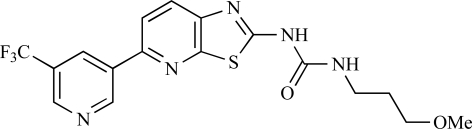	[104]
87	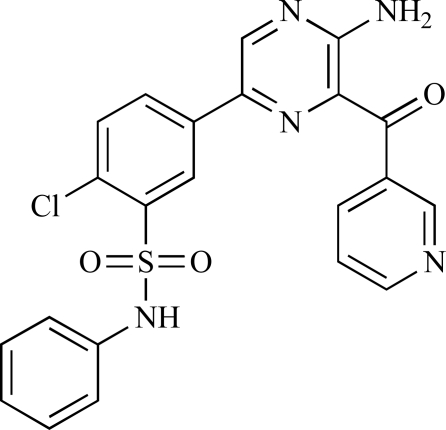	[105]
